# Optimizing recombinant mini proinsulin production via response surface method and microbioreactor screening

**DOI:** 10.1371/journal.pone.0329319

**Published:** 2025-09-08

**Authors:** Esra Ayan, Ali Özhan Aytekin, Ahmet Kati, Hasan Demirci

**Affiliations:** 1 Department of Molecular Biology and Genetics, Faculty of Science, Koç University, Istanbul, Türkiye; 2 Center for Targeted Therapy Technologies, Bogazici University, Istanbul, Türkiye; 3 Experimental Medicine Research and Application Center, University of Health Sciences, Istanbul, Türkiye; 4 Genetics and Bioengineering Department, Engineering Faculty, Yeditepe University, Istanbul, Türkiye; Federal University Dutse, NIGERIA

## Abstract

The increasing demand for efficient recombinant insulin production necessitates the development of scalable, high-yield, and cost-effective bioprocesses. In this study, we engineered a novel mini-proinsulin (nMPI) with enhanced expression properties by shortening the C-peptide and incorporating specific residue substitutions to eliminate the need for enzymatic cleavage. To optimize its production, we applied a hybrid approach combining microscale high-throughput cultivation using the BioLector microbioreactor and statistical modeling via response surface methodology (RSM). Critical medium components were first screened using Plackett–Burman Design (PBD) and refined through Central Composite Design (CDD), identifying glycerol as the most influential factor for yield. Among the four statistically derived formulations, Scenario III demonstrated the highest productivity in the microscale platform (13.00 g/L) and maintained strong performance upon scale-up to a 3-L bioreactor (11.5 g/L). The optimized medium balanced carbon and nitrogen sources to enhance cell viability and maximize protein expression. This study not only confirms the predictive accuracy and scalability of the hybrid optimization system but also introduces a robust production platform for nMPI that can be translated into industrial settings. The workflow presented here can serve as a model for the development of efficient expression systems for complex recombinant proteins in *E. coli.*

## Introduction

The production of recombinant proteins in microbial systems has revolutionized the field of biochemistry, enabling researchers to generate high yields of purified proteins for structural, functional, and pharmaceutical studies [1,2]. Insulin, a peptide hormone critical for glucose homeostasis, remains one of the most widely studied and commercially produced recombinant proteins. Although the overall production strategy—cloning the gene, transforming the host, inducing expression, and purifying the product—appears straightforward in theory [3], practical challenges often emerge. These include limited host cell growth, inclusion body formation, low product yield, and reduced biological activity [4].

Several studies have attempted to overcome these hurdles using diverse expression systems, host strains, and purification protocols [5–8]. However, a key gap in literature remains: the absence of a standardized, scalable, and cost-effective approach for simultaneously evaluating multiple parameters that influence recombinant insulin expression and yield. Inappropriately selected process variables or suboptimal culture conditions can result in significant deviations, thereby complicating production and reducing reproducibility.

A fundamental aspect of recombinant protein production is the choice of host. Among microbial systems, *Escherichia coli* (*E. coli*) remains the preferred host due to its fast growth rate, well-characterized genetics, cost-effective cultivation, and high-density growth capabilities [9–11]. In particular, the T7 expression system offers robust control and high-level expression of recombinant proteins (Studier, 2018). For this study, we selected Rosetta™ 2 (DE3) cells—an engineered *E. coli* strain optimized for the expression of eukaryotic genes by providing tRNAs for rare codons [12]. These strains, induced with isopropyl β-D-1-thiogalactopyranoside (IPTG), are especially compatible with pET vectors for high-yield expression [13].

Our work focuses on expressing a novel mini-proinsulin (nMPI) construct that incorporates a thrombin cleavage site, eliminating the need for additional enzymatic processing. This design simplifies downstream purification while maintaining bioactivity [14]. However, efficient production depends heavily on media composition, induction strategy, and culture parameters, highlighting the need for a systematic approach to process optimization.

To address this, we adopted the Quality by Design (QbD) framework, which has become a cornerstone in bio pharmaceutical development since the U.S. Food and Drug Administration (FDA) introduced the Process Analytical Technology (PAT) guidelines in 2004 [15,16]. Central to QbD is the identification of critical quality attributes (CQAs) and critical process parameters (CPPs), followed by experimental studies to determine their impact on product yield and quality [17,18]. Design of Experiments (DoE) methodologies, including Plackett-Burman and Central Composite Designs, are commonly employed to explore these relationships [19,20].

However, traditional DoE methods may become inefficient when applied to highly complex or multivariate systems due to the large number of required experiments [21,22]. To overcome these limitations, we developed a hybrid microscale screening platform that integrates high-throughput microbioreactor technology (BioLector XT) with statistical modeling tools (Design Expert). This platform allows real-time monitoring of key culture parameters—biomass, pH, dissolved oxygen, and fluorescence—in small volumes (up to 1.5 mL), significantly reducing time and resource demands while preserving experimental resolution [23].

**Fig 1:**
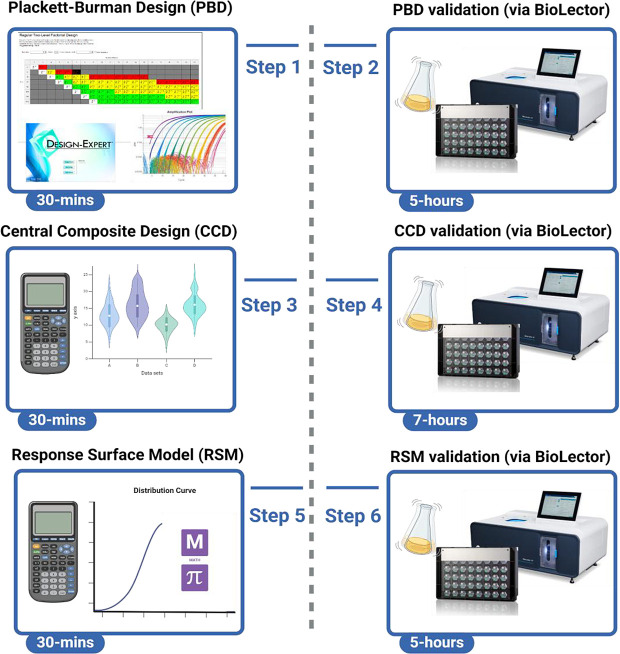
The workflow overview for the straightforward protocol integrated both dry lab (Design-Expert software) and wet lab (microbioreactor) processes. **Step 1:** The protocol began with the setup of 16 initial parameters across 11 certain factors using the Plackett-Burman Design (PBD), which required 30 minutes of dry lab work. **Step 2:** These 16 parameters were then applied to cells within the multi-well micro bioreactor, which spanned 5 hours. **Step 3:** The product yield was analyzed based on PBD results to identify statistically significant factors. Subsequently, the Central Composite Design (CCD) was employed on these significant factors, resulting in 30 parameters for subsequent wet lab trials. This step also took 30 minutes. **Step 4:** The 30 parameters identified by CCD were then applied to cells in the multi-well micro bioreactor, a procedure that required 7 hours. **Step 5:** Product yield was again analyzed to derive statistically significant results from the CCD. The Response Surface Methodology (RSM) was then utilized on these significant results, refining the process to 4 critical parameters for the final wet lab trials. This step requires an additional 30 minutes. **Step 6:** Finally, the 4 refined parameters were applied to cells in the multi-well micro bioreactor, with this final application taking 5 hours.

In this study, we first established a baseline yield of recombinant nMPI using standard LB-based cultivation in Rosetta™ 2 (DE3) cells. The nMPI construct refers to a newly designed, modified mini-proinsulin. It incorporates two main modifications: (i) The native 33-amino acid C-peptide sequence was replaced with a minimal 5-amino acid linker to facilitate proper folding without requiring proteolytic cleavage. This modification allows the molecule to remain functional immediately after expression, eliminating the need for enzymatic processing to remove the C-peptide. (ii) Two amino acid substitutions were introduced to enhance the bioactivity and solubility of the molecule. The first is the replacement of Arg22 in the B-chain with Lys, which renders the molecule more compatible with cleavage by trypsin-like proteases, while still retaining activity even in its uncleaved form. The second is the substitution of Pro28 in the B-chain with Asp, which lowers the isoelectric point of the molecule. This is expected to reduce its tendency to precipitate under physiological conditions and promote a more moderate absorption profile, comparable to that of native human insulin (S1 Fig). The mature form of nMPI, generated through enzymatic cleavage, and its biological activity were previously characterized in detail in our earlier study [24]. nMPI treatment of hiPSCs led to a statistically significant enhancement in cell proliferation, demonstrating a growth-promoting effect similar to that of human insulin (S2 Fig). In addition, the computational simulations supporting the predicted activity and improved solubility of the nMPI are also performed in our another study [25]. Subsequently, we implemented a Plackett-Burman screening design to identify influential culture media components. These were further optimized using a CCD, and results were experimentally validated using the BioLector XT microbioreactor system (**Fig 1**).

Our goal was to develop a user-friendly, scalable, and statistically sound approach to identify optimal conditions for the high-yield production of recombinant miniproinsulin in microbial systems. This study specifically aims to produce and validate a soluble, functionally folded form of nMPI without enzymatic removal of the C-peptide, highlighting the potential for simplified upstream processing.

## Result

We designed and produced a nMPI construct, illustrated by AlphaFold structural predictions in S1 Fig. The engineered molecule preserves the essential domains of insulin—B-chain (blue), A-chain (orange), and a shortened, modified C-peptide segment (gray)—in a compact format. The C-peptide was reduced to a minimal linker sequence (YPGDV), sufficient to permit proper folding while eliminating the need for complex enzymatic processing. Key substitutions, including RB22 to KB22 and PB28 to DB28, were introduced to reduce aggregation propensity, a common issue in insulin formulations [24,26]. These modified residues are highlighted in yellow on the predicted structure. Additionally, a 6xHis tag and a thrombin cleavage site (colored in red) were incorporated at the N-terminus to facilitate purification and enable optional processing. Importantly, the nMPI construct exhibits biological activity comparable to mature insulin [14,25,27] (S2 Fig), bypassing the requirement for tryptic cleavage. This rationally designed molecule serves as the basis for the subsequent optimization of expression and production conditions.

The experimental workflow was structured into six distinct statistical and experimental phases to systematically optimize recombinant mini-proinsulin production (**Fig 1**). **(i)** a PBD was employed to screen and identify significant medium components influencing nMPI yield by evaluating their main effects across a minimal number of experimental runs. **(ii)** The selected PBD matrix was implemented in high-throughput cultivations using the microbioreactor system to generate real-time data on growth and productivity. **(iii)** Statistically significant factors identified in the PBD analysis were further refined using a CCD, allowing for the evaluation of both linear and interaction effects across a broader experimental design space. **(iv)** The CCD matrix was similarly applied in the microbioreactor system to ensure consistency and scalability of the cultivation conditions. **(v)** The results from the CCD experiments were subjected to RSM for modeling the production surface, identifying optimal factor levels, and **(vi)** predict maximum recombinant nMPI yield. This structured design–analyze–refine approach enabled data-driven optimization of production parameters while minimizing experimental burden. Lastly, the experimental design has been performed by large-scale bioreactor to ensure nMPI yielding.

### Initial regression design delivers significant parameters for constructing the model

The study assessed the impact of eleven carefully selected factors, identified through a literature review, on bacterial growth using Design-Expert software (version 7.0.0, Stat-Ease Inc.). A total of 16 experimental runs were conducted using these variables (S1 Table) to assess the growth kinetics of nMPI-producing cells in real-time. For this purpose, the Design-Expert platform was integrated with the BioLector microbioreactor system (Beckman Coulter, Indianapolis, USA), enabling high-resolution, traceable measurements of cell growth dynamics. The PBD, a widely used statistical approach for initial screening experiments, was employed to identify the most effective components for following optimization [28]. The 16 experimental conditions derived from PBD were applied to cultures grown in a multi-well microbioreactor, with each condition performed in triplicate. The final volume in each well was adjusted to 1000 μL. Cells were incubated at 37 °C with constant shaking (800 rpm) for one hour under conditions defined in S2 Table. Throughout the incubation, scattered light intensity was continuously monitored to track cell growth in real time (S3 Fig). Among the factors tested, the concentration of IPTG was included to optimize induction efficiency and minimize production costs. Once cultures reached an optical density (OD_600_) of 0.7–0.8, induction was performed using three IPTG concentrations (0.2 mM, 0.3 mM, and 0.4 mM) by the PBD matrix. After induction, cells were further incubated at 37 °C for one hour. To estimate mini-proinsulin concentration, UV/Vis spectrophotometry at 280 nm was used as a rapid and straightforward method, validated with standard lysozyme solutions. To ensure the reliability and accuracy of the spectrophotometric measurements, a validation test was first performed using manually prepared lysozyme standards at concentrations of 15 mg/mL, 10 mg/mL, 5 mg/mL, and 2.5 mg/mL. Each sample was dissolved in 1 mL of deionized water and directly measured using a Nanodrop spectrophotometer. The absorbance readings precisely matched the calculated concentrations based on the weighed amounts, confirming the instrument’s accuracy.Following this validation step, samples from both nMPI-expressing and non-expressing (control) cultures were analyzed using the same method, with confidence in the device’s performance. Final product concentrations were expressed in grams per liter and confirmed via 20% SDS-PAGE analysis (S3B Fig). Product yield was defined as the response variable and recorded for each experimental condition in S1 Table’s response section.

**Fig 2:**
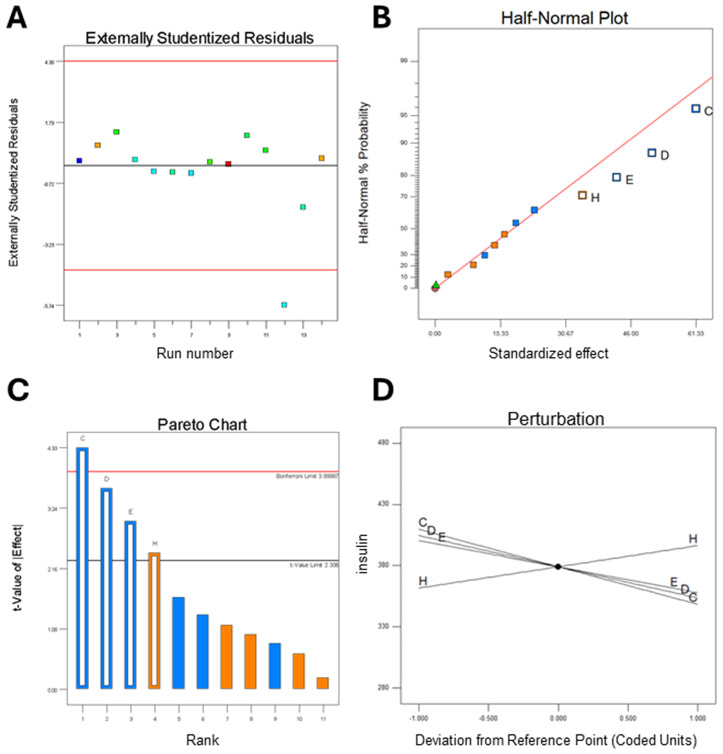
Regression model analysis over residuals, half-normal plot, standardized effects, and factor interactions. (A) Externally studentized residual shows the run number of 12 was out of the limits. (B) The half-normal plot shows that the parameters C, D, E, and H were significant for constructing the model. We can also observe the p-value of parameters in Table 1. (C) Pareto chart shows the Bonferroni and t-value limits based on selected parameters. The limit values were 3.899 and 2.306 of Bonferroni and t-Value, respectively. MgSO_4_ was the only parameter that passed the Bonferroni limit. (D) The perturbation chart also shows that MgSO_4_, glucose, and glycerol have a negative effect on nMPI production; however, yeast has a positive effect (see S3 Table).

ANOVA results for the selected PBD factorial model demonstrated statistical significance, with a *p*-value of 0.05 and a coefficient of determination (*R²*) of 85.37% (**Table 1**, S3 Table). The model identified MgSO₄, glycerol, glucose, and yeast extract as significant contributors, indicating these variables had the most substantial influence on mini-proinsulin production yield (**Fig 2**).

**Table 1:** ANOVA Table Analysis for Selected Factorial Model of Plackett-Burman Design. The analysis of variance (ANOVA) table was obtained for the selected factorial model.

Source	Sum of squares	df	Mean square	F value	p-value Prob > F
**Model**	28155	4	7038,75	11,67	0,0020*
**C-MgSO4**	11285,33	1	11285,33	18,72	0,0025*
**D-Glycerol**	7803	1	7803	12,94	0,0070*
**E-Glucose**	5461,33	1	5461,33	9,06	0,0168*
**H- Yeast**	3605,33	1	3605,33	5,98	0,0402*
**Curvature**	2674,71	1	2674,71	4,44	0,0683 ns
**Residual**	4823,5	8	602,94		
***Lack of Fit***	4783	7	683,29	16,87	0,1854 ns
***Pure Error***	40,5	1	40,5		
**Cor Total**	35653,21	13			
**Final Statistics:**					
**R-squared**		0.8537		**Std. Dev.**	24.55
**Adj R-squared**		0.7806		**Mean**	373.36
**Pred R-squared**		0.6012		**C.V. %**	6.58
**Adeq Precision**		11.799		**PRESS**	14218.16

Analysis of externally studentized residuals identified an outlier in run number 12, which exceeded acceptable limits (**Fig 2A**). Despite this anomaly, the regression model remained statistically robust, with a coefficient of determination (*R²*) greater than 0.85 (**Table 1**), a threshold commonly considered acceptable in biological studies [29]. The half-normal plot is a diagnostic tool used to assess the normality assumption of a model by evaluating the distribution of residuals [30]. In **Fig 2B**, the linear alignment of residuals suggests that they follow a normal distribution. Deviations from this linear trend would have indicated violations of the normality assumption. Furthermore, the half-normal plot effectively visualized the standardized effects of independent variables, highlighting MgSO₄, glycerol, glucose, and yeast extract as the most significant factors influencing the response. To further confirm variable significance, a Pareto chart was created to display the standardized effects of each factor [31]. Threshold values for significance were set at 3.899 for the Bonferroni limit and 2.306 for the t-value. Among all tested parameters, only MgSO₄ exceeded the Bonferroni threshold, indicating a particularly strong effect (**Fig 2C**). However, glycerol, glucose, and yeast also showed significant impacts, supporting their inclusion in the model as significant contributors. A perturbation plot was used to compare the relative effects of individual variables around a central point in the design space [32]. This analysis confirmed that MgSO₄, glucose, and glycerol negatively influenced nMPI production, suggesting that lower concentrations of these components may enhance yield. In contrast, yeast extract exhibited a positive effect, indicating that increasing its concentration could improve nMPI output (**Fig 2D and** S3 Table).

### Quadratic modeling identifies glycerol as a key modulator in multivariate culture

To further optimize the statistically significant factors identified in the initial screening—MgSO₄, glycerol, glucose, and yeast extract—a CCD was applied, generating 30 experimental conditions (S4 Table). These conditions were tested using the microbioreactor platform. Throughout all experiments, baseline medium components were kept constant, including 10 mM KCl, 15 mM MgCl₂, 15 mM KH₂PO₄, and 10 mM thiamine. When cultures reached an OD₆₀₀ of 0.7–0.8, induction was carried out with 0.25 mM IPTG for each condition, followed by a 1-hour incubation at 37 °C (S4A Fig). Final nMPI concentrations were quantified in grams per liter using UV/Vis spectrophotometry at 280 nm and further validated by 20% SDS-PAGE analysis (S4B Fig). nMPI yield served as the response variable and was recorded for each run in the CCD matrix (S4 Table, *response* section). After testing, we created a quadratic model to show how the variables relate to the response. This model helps us predict and confirm the best conditions. The model demonstrated strong statistical significance, particularly for the effect of glycerol, with a *p*-value of 0.0037 (**Table 2**), supporting its reliability. The model did not fit the data well, as shown by a statistical value of **p* *= 0.4479. This means that the errors in predictions were small and did not significantly affect the model’s accuracy (**Table 2**). Although the *R²* value of the model was moderate (0.6866), the residual deviation (3.3%) remained well within the acceptable range of 5–10% for biological regression models [29]. This level of accuracy was consistent with our experimental validation, as reflected in the outcomes summarized in **Table 3**.

**Table 2:** The analysis of the variance table is used to perform ANOVA for a Response Surface Reduced Quadratic Model.

Source	Sum of squares	df	Mean square	F value	p-value Prob > F
**Model**	201.91	8	25,24	5,31	0.0010*
**A-Glucose**	1,25	1	1,25	0,26	0.6139
**B-Glycerol**	93,10	1	93,10	19,58	0.0002*
**C- Yeast**	2,82	1	2,82	0,59	0.4502
**D-MgSo4**	4,04	1	4,04	0,85	0.3671
**AB**	7,29	1	7,29	1,53	0.2293
**BC**	4,20	1	4,20	0,88	0.3579
**BD**	4,84	1	4,84	1,02	0.3245
**B** ^**2**^	84,38	1	84,38	17,74	0.0004*
**Residual**	99,86	21	4,76		
**Lack of Fit**	78,33	16	4,90	1,14	0.4833 ns
**Pure Error**	21,53	5	4,31		
**Cor Total**	301,77	29			
**Final statistics:**					
**R-squared**			0.6866	**Std. Dev.**	2.23
**Adj R-squared**			0.5216	**Mean**	9.46
**Pred R-squared**			0.1663	**C.V. %**	23.59
**Adeq precision**			7.861	**PRESS**	351.97

**Table 3:** The forecasted optimum conditions for achieving the maximum production of nMPI.

Scenario	Glucose (mM)	Glycerol (%)	Yeast (g/L)	MgSO_4_ (mM)	Predicted Insulin (g/L)	Observed Insulin (g/L)	Deviation (%)
**1**	14.58	1	2.51	15	13.92	11.87	14.8
**2**	15	1	2.5	6.73	13.79	11.54	16.3
**3**	**8.78**	**1**	**2.5**	**15**	**13.44**	**13.00**	**3.3**
**4**	15	1	3.13	10.62	13.21	12.33	6.7

**Fig 3:**
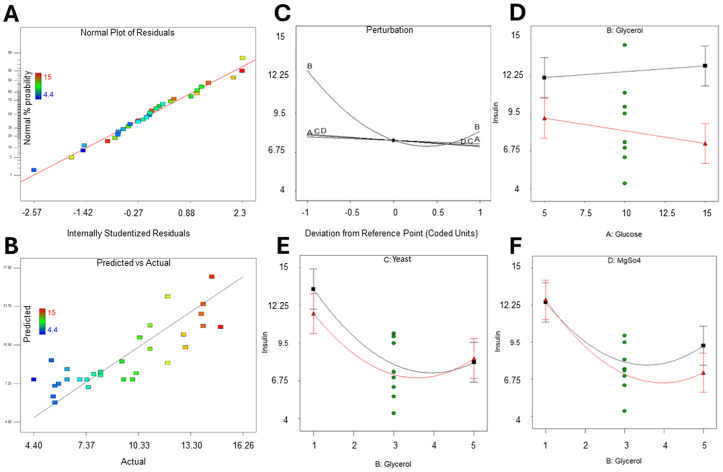
Evaluation of model sensitivity and parameter interactions in nMPI production. (A) Internally studentized residuals show the correction of chosen parameter concentration. All runs were close to the probability line, which means the selected concentrations were correct for optimization. (B) The nMPI ranges in the Predicted vs. Actual chart. Low concentrations of nMPI shifted a bit from the actual data. The other concentration ranges distribute homogeneously around the line. (C) Perturbation plot. Glycerol significantly affects nMPI production, while glucose, yeast, and MgSO_4_ have no significant impact on these concentration ranges. (D, E, F) The interactions of parameters. (D) Glucose and glycerol had no interactions in these concentration ranges. However, (E) glycerol interacted with yeast at high concentrations, while at low concentrations (F), there was an interaction with MgSO_4_.

We looked at studentized residuals to check if the selected factor concentrations were suitable (**Fig 3A**). Most data points closely followed the probability line, which shows that the chosen concentrations were right for the optimization process. In the Predicted vs. Actual plot (**Fig 3B**), nMPI concentration values should ideally be close to the diagonal reference line, which shows high predictive accuracy. The model works well overall, but there are some small deviations at lower nMPI concentrations, indicating slight inaccuracies in these predictions. On the other hand, medium to high nMPI concentrations show a more consistent distribution around the reference line, proving that the model performs strongly and reliably in this range. These results show that the model reliably predicts moderate to high nMPI yields. While it performs slightly worse at low concentrations, this does not significantly affect its overall ability to make accurate predictions. Additionally, a perturbation plot was used to examine the model’s sensitivity to individual factors (**Fig 3C**). This analysis revealed that glycerol (factor B) had a particularly strong influence on the response variable, further highlighting its critical role in nMPI production. Instead, glucose, yeast, and MgSO_4_ (factors A, C, and D, respectively) have much less impact compared to factor B, which is important for boosting nMPI production (**Fig 3C**). The data in **Table 2** and S5 Table support this finding. Testing offers that factors A, C, and D have little effect on the concentrations we tested. We looked closely at how these factors interact, which we show in **Figs 3D**, **3E, and 3F**. The results indicate that, at the tested concentrations, glucose does not interact with glycerol (**Fig 3D**). However, glycerol does interact significantly with other components. It interacts with yeast at higher concentrations (**Fig 3E**) and with MgSO_4_ at lower concentrations (**Fig 3F**). Understanding these interactions is important for optimizing the medium composition. It suggests that we should adjust the amounts of yeast and MgSO_4_ based on the level of glycerol to create the best conditions.

### The adapted hybrid approach made the finest recipe for mini-proinsulin production

**Fig 4:**
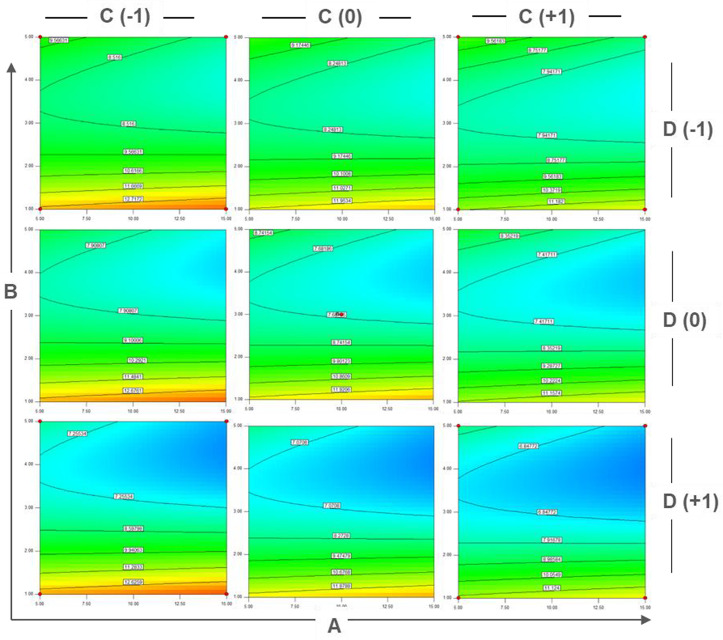
Factorial contour plots of Central Composite Design. A representative plot is an effective visualization tool, capturing the intricate relationship between the independent variables and the resulting nMPI yield. The intentional manipulation of these independent variables within a predetermined range allowed for systematically exploring their collective impact on nMPI production. Elevating glycerol (B) levels adversely affects nMPI efficacy, whereas increasing glucose (A) levels positively influences nMPI yield. Maintaining yeast (C) at lower levels contributes positively to nMPI yield. Conversely, increasing MgSO4 (D) concentrations negatively impacts nMPI efficacy. The best factorial composition is characterized by elevated glucose (A) and lower levels of yeast (C) and MgSO4 (D), giving the highly yielded nMPI concentration of around 13 mg/ml (*top left corner*).

**Fig 5:**
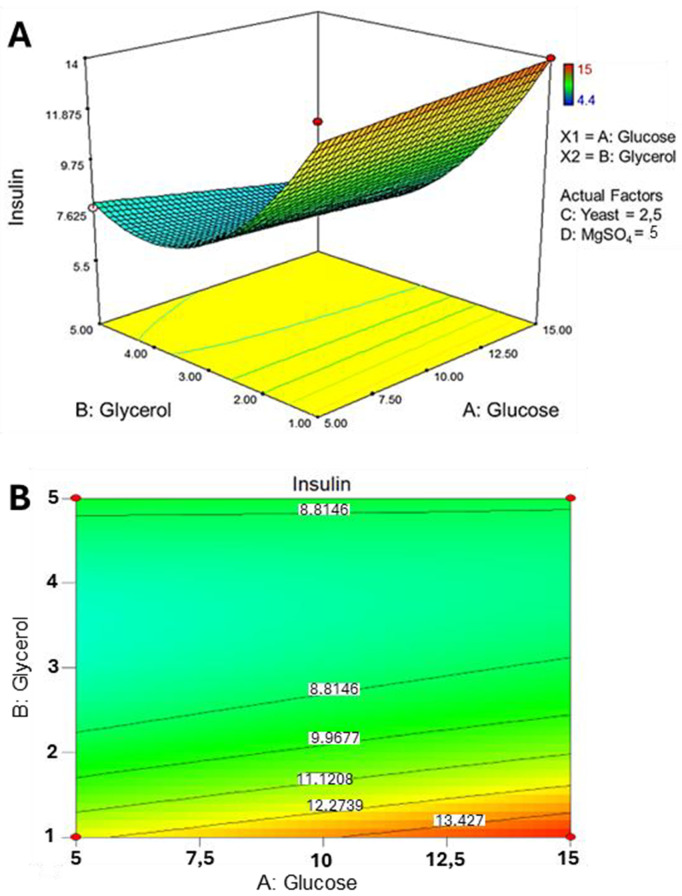
Illustrating the interactive effects of glucose, glycerol, MgSO_4_, and yeast concentrations on the efficiency of nMPI production while keeping temperature and pH constant. (A) The 3D response surface plot indicates that 1% glycerol and 15 mM glucose positively affect nMPI production in the presence of 2.5 g/L yeast and 5 mM MgSO4, providing around 13 g/L nMPI efficacy. (B) 2-factorial contour plots validate that a lower level of glycerol and a higher level of glucose provides a high-yield product.

System calculated factorial contour plots (**Fig 4**) and response surface analysis (**Fig 5A**) to better understand how different factors affect nMPI yield. The two-dimensional contour plots (**Fig 4**) and the three-dimensional response surface plot (**Fig 5A**) show how the independent variables relate to nMPI yield. We changed these independent variables within a set range to see how they work together to influence nMPI production. The contour plots (**Fig 4**) give useful insights into how different factors interact. For example, higher levels of glycerol and glucose, along with lower amounts of MgSO_4_ and yeast, reduce nMPI yield from 12.7172 mg/ml to 9.5663 mg/ml (**Fig 4, top left corner**). Also, increasing glycerol and yeast at higher levels of MgSO_4_ and glucose decreased nMPI yield from 11.124 mg/ml to 6.84772 mg/ml (**Fig 4, bottom right corner**).

We calculated a 3D response surface plot (**Fig 5**) to clarify how different variables interact with each other. This plot clearly showed how glucose, glycerol, MgSO_4_, and yeast concentrations affect nMPI production in 3D rendering (**Fig 5A**). We found that using a higher glucose concentration in the presence of 2.5 g/L of yeast and 5 mM of MgSO_4_ led to increased nMPI yield (**Fig 5A**). This combination produced more nMPI than any other variable we tested.

**Fig 6:**
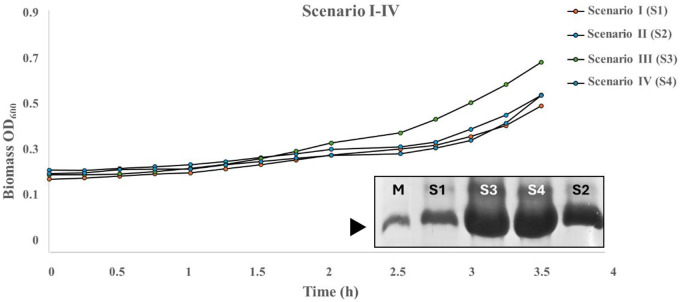
Production efficacy of nMPI and analytical characterization. Growth kinetics of 4 scenarios obtained by RSM using the multi-well micro bioreactor. Each parameter was triplicated. 20% SDS-PAGE analysis for determining nMPI efficacy. Each band indicates each scenario’s protein yield, with no amount of equalization performed to assess direct protein efficacy. The original gel image is in the supplementary file (S4A Fig). M: marker.

Four distinct scenarios were statistically derived and subjected to experimental validation based on these findings. The experimentation was performed using the micro bioreactor (1 mL), with each scenario being replicated three times to ensure the reliability and accuracy of the results. All components of the baseline medium were kept constant during the experiments, including 10 mM KCl, 15 mM MgCl₂, 15 mM KH₂PO₄, and 10 mM thiamine. Despite the absence of substantial discrepancies among the scenarios, noteworthy that scenario III—characterized by a glucose concentration of 8.78 mM, glycerol concentration of 10 g/L, yeast at 2.5 g/L, and MgSO_4_ concentration of 15 mM—exhibits the slightest deviation, and the highest nMPI production compared to the further scenarios (**Table 3**). The final nMPI concentrations cultivated by microbioreactor (**Fig 6**) were quantified using UV/Vis spectrophotometry at 280 nm (**Table 3**) and confirmed by 20% SDS-PAGE analysis (**Fig 6**)

**Fig 7:**
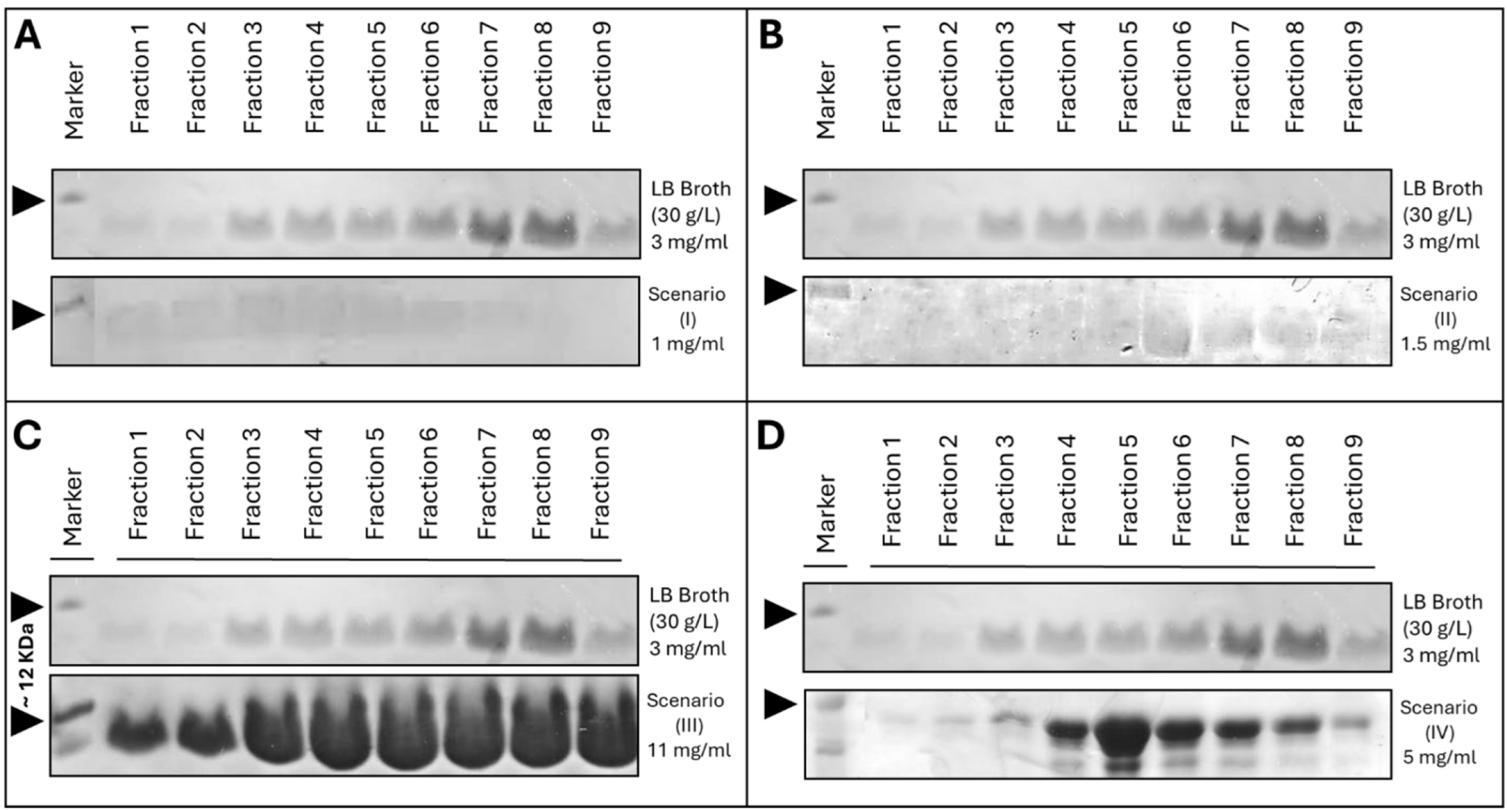
SDS-PAGE analysis of nMPI production under large-scale (3L) cultivation conditions across four optimized media scenarios compared to standard LB medium. Panels A–D compare the elution fractions [1–9] of purified nMPI expressed in 3-L bioreactors using LB broth (30 g/L) and each of the four optimized media formulations (Scenarios I–IV), as defined in Table 3. (A) Scenario I yielded 11.87 mg/mL in microbioreactor assays but only ~1 mg/mL in 3-L scale. The protein bands in all fractions appear faint, indicating poor scalability. (B) Scenario II showed a predicted yield of 11.54 mg/mL in microscale, but only ~1.5 mg/mL was obtained in the 3-L bioreactor. Fractions remained faint and inconsistently distributed. (C) Scenario III demonstrated strong scalability: although the predicted yield was 13.00 mg/mL, the actual production in the 3-L bioreactor reached 11.5 mg/mL. Bands are clearly visible and evenly distributed across all fractions, indicating robust expression and purification efficiency. (D) Scenario IV showed moderate scalability, with an observed yield of ~5 mg/mL compared to 12.33 mg/mL in the microscale. Bands are moderately intense and visible across mid-fractions. In all panels, the top gels show production from LB broth, which served as the baseline control. The lower gels in each panel correspond to the scenario-specific media. Arrowheads indicate the expected molecular weight region for nMPI. The original gel images are in the supplementary file (S4B-F Fig*)*. M: marker (~12 kDa).

To further evaluate the scalability and practical applicability of the optimized conditions identified in the micro-scale experiments, all four statistically derived scenarios were independently tested in 3-liter bench-top bioreactors. Each condition was scaled up under controlled parameters to validate whether the nMPI yields observed in the multi-well microbioreactor could be reliably reproduced in larger volumes. In addition, a control experiment was performed using a standard LB medium formulation (30 g/L LB broth) under identical fermentation and induction (0.25 mM IPTG) conditions to benchmark the performance of the optimized media. For all the scenarios, all the ingredients in the basic mixture stayed the same during the experiments. This included 10 mM of KCl, 15 mM of MgCl₂, 15 mM of KH₂PO₄, and 10 mM of thiamine. The final nMPI concentrations were quantified using UV/Vis spectrophotometry at 280 nm and confirmed by 20% SDS-PAGE analysis (Fig 7 and S5 Fig).

SDS-PAGE analysis of elution fractions from large-scale cultures revealed clear differences in nMPI expression levels across the conditions (Fig 7). Scenario I, despite an observed yield of 11.87 mg/mL in the microbioreactor, resulted in only ~1 mg/mL in the 3-L bioreactor, with faint and diffuse bands observed across fractions (Fig 7A). Similarly, Scenario II showed limited scalability, producing ~1.5 mg/mL in the bioreactor versus the observed 11.54 mg/mL in the microbioreactor, again with weak and inconsistently distributed bands (Fig 7B). In contrast, Scenario III demonstrated prominent scalability: although the observed yield was 13.00 mg/mL in microbioreactor, the actual production reached 11.50 mg/mL in large-scale format, with strong and well-resolved bands across all fractions (Fig 7C). Scenario IV showed intermediate performance, yielding ~5 mg/mL compared to an observed 12.33 mg/mL in microbioreacot, with moderate band intensity (Fig 7D**).** Across all scenarios, LB broth (30 g/L) produced ~3 mg/mL of nMPI and served as a baseline reference. These results confirm that Scenario III not only offers the best predictive-to-observed yield correlation in micro- and large-scale production but also exhibits superior protein expression consistency.

## Discussion

The rising demand for efficient recombinant insulin production presents several challenges. These challenges include optimizing cell growth under high expression conditions, enhancing vector systems, engineering smarter designs, and adjusting gene and insulin sequences to align with the host’s biosynthesis machinery. There is a need for straightforward and highly efficient methods that can be monitored in real-time and are applicable at both laboratory and industrial scales. These methods should improve production efficiency by integrating statistically significant parameters within a semi-automated system.

In this study, we utilized a hybrid micro-screening system to produce high-yield modified mini-proinsulin. This nMPI has been re-engineered to enhance production efficacy by shortening the C-peptide and by substituting the residues R22B with K22B and P28B with D28B. Furthermore, the previously necessary enzymatic cleavage of the nMPI sequence has been modified to eliminate this requirement, allowing for the immediate production of active mini-proinsulin upon completion [14] (S1**-**S2 Fig). Through the application of the hybrid screening method, we achieved production levels of up to 13 mg of nMPI per 1 mL, with a large-scale output of 11.5 mg/mL.

The hybrid system integrates a real-time traceable smart plate and utilizes RSM as illustrated in **Fig 1**. The main objective is to optimize the composition of various cultivation media on a micro-scale platform before transitioning to lab-scale and industrial insulin production. Similar to other natural processes, bacterial growth rates are influenced by various factors. Key parameters include the type and concentration of carbon and nitrogen sources, pH conditions, and the presence of trace elements, all of which have garnered significant attention. Identifying and optimizing these factors present considerable financial and economic challenges [33]. To tackle these challenges, combining experimental micro-bioreactors (BioLector) with computational RSM offers a robust and practical method for optimizing insulin bioprocesses at both lab and industrial scales. This integrated approach yields reliable results while minimizing the time and resources needed (**Fig 1**). We prefer to employ RSM for experimental screening, as several studies have successfully used this statistical analysis to enhance high-yield recombinant protein production in *E. coli.* These studies have focused on optimizing various culture media, which encompass different protein types, design methodologies, factors under evaluation, and the resulting optimization outcomes [34–38].

Several studies have focused on utilizing the multi-well microbioreactor platform [39–42]. This screening system is designed to enhance the specific productivity of the *E. coli* T7 expression system through phosphate limitation [43]. Additionally, it has demonstrated effectiveness in real-time lipid production and monitoring growth in Y. lipolytica [44]. The literature highlights the transferability and comparability of results between the BioLector system and fully controlled bioreactor systems operating in fed-batch mode, especially at moderate to high cell densities [45]. Various studies have shown the BioLector system’s efficacy in screening optimal growth conditions and engineered strains before scaling up [46]. Using a hybrid approach that combines RSM and BioLector, researchers have successfully performed high-throughput optimization of medium components and culture conditions to efficiently produce the lipopeptide pseudofactin by Pseudomonas fluorescens BD5 [47]. The key distinction of our study compared to previous research is the media optimization performed with a newly designed and modified mini-proinsulin, nMPI. This approach results in improved efficiency and significantly shorter timeframes than those achieved with the hybrid system. While many studies have utilized this hybrid system, our research uniquely aims to achieve the highest possible nMPI production, reaching up to 13 mg/ml in micro-, 11.5 mg/ml in large-scale, and ensures that this mini-proinsulin is immediately ready for use.

The results of this study, based on RSM, indicate that glycerol concentration is the most critical factor influencing nMPI production. Higher glycerol concentrations are directly associated with increased nMPI production. This finding aligns with previous research that highlights the positive effect of glycerol on *E. coli* growth, particularly in promoting anaerobic fermentation (Dharmadi et al., 2006). However, when comparing the combined use of glycerol as a carbon source with IPTG as an inducer, the study determined that the optimal glycerol concentration should not exceed 1% (v/v) in the presence of IPTG. This observation is consistent with other studies that report a decrease in cell yield as glycerol concentration increases [48]. While glycerol is beneficial, it is essential to maintain the optimal concentration of up to 1% to avoid diminishing returns on cell growth and nMPI production, as illustrated in **Figs 4 and 5**. Among various critical factors, glucose and MgSO_4_ have been established as vital for *E. coli* growth [49]. The positive effects of limited glycerol may stem from the harmful impact of glucose when used as a carbon source, especially under conditions of low nitrogen availability [50,51]. This observation aligns with our final media component (**Table 3**). Harsh inducers like IPTG can trigger cell stress responses and reduce growth rates. Gentler inducers have been employed to address these issues. When combined with glucose, glycerol might exhibit less catabolite repression against IPTG. This combination yields positive results in recombinant protein production, suggesting that a moderate presence of glycerol serves as a more suitable carbon source, enhancing cell viability and productivity [52].

The metabolic breakdown of the carbon source results in the accumulation of acidic by-products, primarily acetate, in the culture medium. These acidic conditions can significantly impede cell growth and reduce recombinant protein production. To counter this issue, adding yeast extract to the culture medium helps mitigate acidification by balancing the elevated ammonia levels produced during metabolic breakdown [53,54]. Among the scenarios analyzed (**Table 3**), Scenario III represents the optimal formulation of the culture medium, featuring a balanced combination of lower concentrations of yeast, glycerol, glucose, and 15 mM MgSO_4_. This specific combination is likely to enhance cell growth and delay the onset of the death phase, making it well-suited for improving cellular dynamics and extending cell viability.

All findings indicate that keeping moderated glycerol levels, with the appropriate media composition and precise induction timing, effectively redirected cellular resources towards protein expression rather than biomass production during cultivation. This might be the first time the combined effect has been observed in an IPTG-inducible nMPI expression system. Additionally, this study by applying the introduced high-throughput hybrid experimentation approach, could play a crucial role in optimizing future production studies involving recombinant *E. coli* strains.

## Conclusion

This study introduces a comprehensive hybrid strategy for optimizing the culture conditions of a nMPI using the *E. coli* Rosetta™ 2 expression system. By combining real-time microscale screening with the BioLector microbioreactor and employing statistical design-of-experiments approaches (including PBD and CDD) along with RSM, we successfully identified an efficient medium formulation. This formulation maximizes mini-proinsulin yield while minimizing experimental complexity. Among the tested formulations, Scenario III proved to be the most effective, achieving a predicted yield of 13.44 g/L and an experimentally validated yield of 13.00 g/L in the microbioreactor, with only a 3.3% deviation. Notably, this formulation demonstrated strong scalability; when adapted to 3-liter stirred-tank bioreactors, it maintained a high production level of 11.5 g/L, surpassing other scenarios and confirming the model’s predictive capability across different scales. Our findings emphasize the importance of glycerol as a crucial modulatory factor, with moderate concentrations (1%) enhancing nMPI expression without inhibiting growth. Additionally, a balanced combination of glucose, MgSO₄, and yeast extract in the optimized medium contributed to prolonged cell viability and effective resource allocation toward recombinant protein production instead of biomass accumulation. The redesigned nMPI construct, which eliminates the need for enzymatic cleavage, combined with this optimized medium, facilitates a highly productive and scalable system suitable for industrial applications. In conclusion, this work establishes a transferable and predictive framework for micro-to-large scale optimization in recombinant nMPI production. The hybrid strategy outlined here can serve as a blueprint for accelerating the development of high-efficiency bioprocesses in synthetic biology and industrial biotechnology, particularly for complex recombinant proteins.

## Methods

### Microorganisms, culture media, chemicals, BioLector and Software

We transformed the *E. coli* BL21 Rosetta 2 strain using the pET28a(+) vector, which contains a nMPI construct with a removable 6xHis-tag at the start. We sourced all necessary chemicals from trusted suppliers, including Merck and Sigma. Our team provided the KUYBIIGM-Ladder protein weight marker. For designing and analyzing our optimization experiments, we used the Design-Expert 7.0.0 software from Stat-Ease, Inc. in Minneapolis, MN, USA. We conducted screening experiments with the BioLector XT Microbioreactor in Indianapolis, focusing on statistically determined parameters.

### Media optimization

We started by screening different factors using the Plackett-Burman Design (PBD) method to find the most important variables. After that, we used the Central Composite Design (CCD) method from Response Surface Methodology (RSM) to optimize the levels of these key variables. All experiments took place in 2 mL volume wells in the BioLector XT Microbioreactor. Each well contained 1 mL of the specific culture medium, which we prepared based on certain design points and inoculated with an overnight seed culture. We grew the inoculated cells for 4 hours in each well, stirring continuously at 800 rpm and maintaining a constant temperature of 37°C. We used 48-well FlowerPlates from mp2-labs in Baesweiler, Germany. We monitored growth by measuring scattered light intensities in real time. Following bacterial growth, the cells harboring the nMPI plasmid were harvested and pelleted by centrifugation (3500 rpm x 10 mins). The resulting pellets were solubilized overnight at room temperature on a shaker using 250 μL of reducing solubilization buffer containing 25 mM Tris-HCl (pH 9.0), 5 mM 2-mercaptoethanol, and 8 M urea. Complete dissolution of the pellets was observed under these conditions. To determine the final nMPI concentration, we used a UV/Vis spectrophotometer set to a wavelength of 280 nm. The absorbance of the non-induced sample (lacking nMPI expression; *before induction*) was subtracted from that of the induced sample (overexpressing nMPI; *after induction*) to account for background signal and isolate the contribution of the recombinant protein. We analyzed the results further using 20% SDS-PAGE (Sodium Dodecyl Sulfate Polyacrylamide Gel Electrophoresis). Raw versions of all gel images are presented in S1 File_raw_image.

### Screening of factors using the Plackett-Burman factorial design approach

We studied eleven factors that could affect our experiment. These factors included different sources of nitrogen and carbon, pH levels, various media concentrations, and the presence of salts and metal ions. We designed the experiment using a Plackett-Burman factorial design, which involved sixteen tests conducted with DoE software. After performing the BioLector experiments, we analyzed the results statistically. We presented our findings, including model validation and the importance of each variable, in tables that showed ANOVA and fit statistics. We identified significant variables (with p-values less than 0.05) by looking at the ANOVA table and tools like the Half-normal plot, Pareto chart, and Perturbation chart. These methods helped us determine which factors had a major impact on the results of our experiments.

### Optimizing factors using response surface methodology

Based on the PBD results, we selected four important factors for further optimization using CCD, which required thirty experimental runs. We prepared the culture media for each run using the specific composition of the selected factors, following the design points in BioLector. We also added constant values for other media components that were not part of the model, based on nMPI results measured in mg/ml from the PBD design and SDS-PAGE results. We supplied the concentration corresponding to the highest response for less important variables, along with the −1 level of variable factors that were insignificant. After conducting the experiments in BioLector, we analyzed the responses using different models. We chose the best model based on validation parameters shown in the ANOVA table, fit statistics, and diagnostic analysis. The DoE software created a perturbation chart, and we illustrated the effects of each significant independent and dependent variable on the response using contour and 3D plots. Finally, the software generated four scenarios for optimal points based on the regression equation. We tested the predicted design scenarios with the highest desirability in triplicate using the BioLector XT microbioreactor. The optimal medium identified was Scenario-3, which yielded 13 mg/ml with a 3.3% deviation, and is ready for scale-up optimization. Raw versions of all gel images are presented in S1 File_raw_image.

### Large-scale production

Initially, stock cells containing nMPI genes were cultured overnight in 150 mL of LB medium (30 g/L), supplemented with 50 µg/mL kanamycin and 35 µg/mL chloramphenicol, at a temperature of 37°C and a shaking speed of 110 rpm using a New Brunswick Innova 4430R incubator. Following this, a 150 mL cell culture was transferred to a 6 L bioreactor (Braun Biotech Sartorius 6L Jacketed Glass Vessel Bioreactor) containing the same antibiotics and 4 mL of antifoam. The main cultivation process was conducted at 37°C with an initial culture volume of 3 L. The air flow rate was set at 15 L/min, and the stirrer speed was maintained at 800 min ⁻ ¹. Thermal mass flow meters (Bronkhorst High-Tech B.V., Ruurlo, The Netherlands) were used to mix air and oxygen. The concentration of dissolved oxygen was monitored using two polarographic electrodes (Ingold, Germany) and was kept at 30% of air saturation by adjusting the stirrer speed and aeration rate. Pure oxygen was added to the inlet air as needed. The pH was maintained at 6.8 by adding aqueous ammonia (25% w/w), with all controls managed by the bioreactor’s process control system. When the optical density reached 0.7, protein production was induced by adding 0.25 mM IPTG to the cultured cells, followed by a three-hour incubation at 37°C and a shaking speed of 250 rpm. After the incubation period, cell pellets were harvested by centrifugation at 3500 rpm for 45 minutes using a Beckman Allegra 15R centrifuge, and the collected pellets were stored in a −80°C freezer.

### Solubilization, purification, sulfitolysis and refolding

The downstream process of large-scaled nMPI was performed following Ayan et al. study (Ayan et al., 2025). Briefly, to partially purify and completely solubilize inclusion bodies, 1 gram of cell pellets containing nMPI proteins were resuspended in 10 mL of lysis buffer composed of 25 mM Tris-HCl (pH 8.0) and 5 mM Ethylenediaminetetraacetic acid (EDTA). The bacterial cells were lysed using sonication, and the resulting cell debris was subjected to low-speed centrifugation at 6000 × g for 7 minutes at 4°C. The obtained pellets were then resuspended in wash buffer A, which contained 25 mM Tris-HCl (pH 8.0), 5 mM EDTA, 0.1% (v/v) Triton X-100, and 1 M urea. This resuspension was sonicated for 30 seconds in an ice bath, followed by centrifugation at 8000 × g for 20 minutes at 4°C. To further isolate the inclusion bodies, the debris was resuspended at a concentration of 0.1 g/mL in a binding buffer consisting of 25 mM Tris-HCl (pH 8.4), 5 mM 2-mercaptoethanol (BME), and 8 M urea. Centrifugation at 10,000 × g for 30 minutes at 4°C enabled the recovery of inclusion bodies containing the fusion proteins. The resulting supernatants were filtered through a 0.2-micron Millipore filter. Later, sulfitolysis of nMPI was performed at 25°C for 4 hours using 200 mM sodium sulfite (Na₂SO₃) and 20 mM sodium tetrathionate (Na₂S₄O₆). Manual gravity Ni-NTA affinity chromatography was employed next, which involved column equilibration with one column volume of a buffer consisting of 25 mM Tris-HCl (pH 8.0), 5 mM BME, and 8 M urea. Elution was carried out using a buffer of 25 mM Tris-HCl (pH 8.0), 5 mM BME, 8 M urea, and 250 mM imidazole. The purity of the nMPI sample was assessed at each stage through 20% SDS-PAGE gel analysis and Coomassie staining. High-purity fractions were collected and filtered through a 0.2-micron Millipore filter. The purified nMPI (at a concentration of 0.5 mg/mL) was then dialyzed against 25 mM Tris-HCl (pH 9.00) containing 8 M urea and 0.5 mM EDTA at 4°C. After centrifugation at 10,000 × g for 30 minutes at 4°C to remove potential aggregates, the resulting mixture was dialyzed in a refolding solution of 0.1 M Gly/NaOH (pH 10.5), 0.5 M urea, 0.5 mM EDTA, and 5% glycerol, stirring at 15°C overnight. Finally, the refolded nMPI was dialyzed against 25 mM Tris-HCl (pH 8.0) containing 5% glycerol for 18 hours.

### Proliferative effects of insulin monomers in hiPSC culture systems

Human-induced pluripotent stem cells (hiPSCs) were generated from peripheral blood mononuclear cells (PBMCs) through a non-integrative Sendai viral vector system that delivers Yamanaka reprogramming factors. After successful reprogramming, cell colonies were expanded on Matrigel-coated plates utilizing mTeSR1 medium (StemCell Technologies) and were regularly subcultured with 0.5 mM EDTA. Before experimental procedures, all hiPSC lines were cryopreserved and later re-adapted to the TeSRE8 culture medium for at least two weeks to guarantee stability. The nMPI, created as detailed in the Methods section, was used for comparative evaluations. For treatment assays, hiPSCs were cultured in a defined E8 medium formulation supplemented with essential additives, including 100 U/ml penicillin, 100 µg/ml streptomycin, 20 mg/ml transferrin, 3 mM sodium selenite, 100 mg/ml ascorbic acid, 1 mg/ml FGF2-G3, and 0.5 µg/ml TGF-β3. Either 20 mg/ml of the new nMPI analog or corresponding concentrations of commercial recombinant insulin were added to the medium. Cultures were kept at 37°C in a humidified environment with 5% CO₂. Cell proliferation patterns were evaluated at designated time points—Days 3 and 7—using the Mateo TL Digital Transmitted Light Microscope. Quantitative findings indicated that nMPI had a proliferative influence on hiPSCs similar to that of human insulin, underscoring its potential as a functionally similar analog.

## Supporting information

S1 Fig Alpha-fold prediction of novel designer fast-acting mini-insulin and detailed sequence. (PDF)

S2 Fig Comparison of cell growth responses in hiPSCs treated with nMPI and human insulin over time. (PDF)

S3 Fig Production efficacy of modified proinsulin and analytical characterization. (PDF)

S4 Fig Production efficacy of modified proinsulin and analytical characterization. (PDF)

S5 Fig Original 20% SDS-PAGE analysis of nMPI expression across microscale and large-scale cultivation conditions. (PDF)

S1 Table The 16 parameters created by PBD analysis of eleven factors and insulin efficiencies were obtained due to experimental adaptation. (PDF)

S2 Table Multi-well micro bioreactor parameters for achieving the highly-yielded biomass in a shorten time. (PDF)

S3 Table Coded and actual values of independent variables. (PDF)

S4 Table The 30 parameters created by CCD analysis of statistically significant factors and insulin efficiencies were obtained due to experimental adaptation. (PDF)

S5 Table Coded values of independent variables. (PDF)

S1 Raw Images Uncropped and unadjusted gel image of supplementary figures. (PDF)
