# Supplementary material for: Optimizing recombinant mini proinsulin production via response surface method and microbioreactor screening
Source: PLoS One. 2025 Sep 8;20(9):e0329319. doi: 10.1371/journal.pone.0329319 (PMC12416663; doi:10.1371/journal.pone.0329319)
Supplement: S1 Fig — (PDF) [file pone.0329319.s001.pdf]

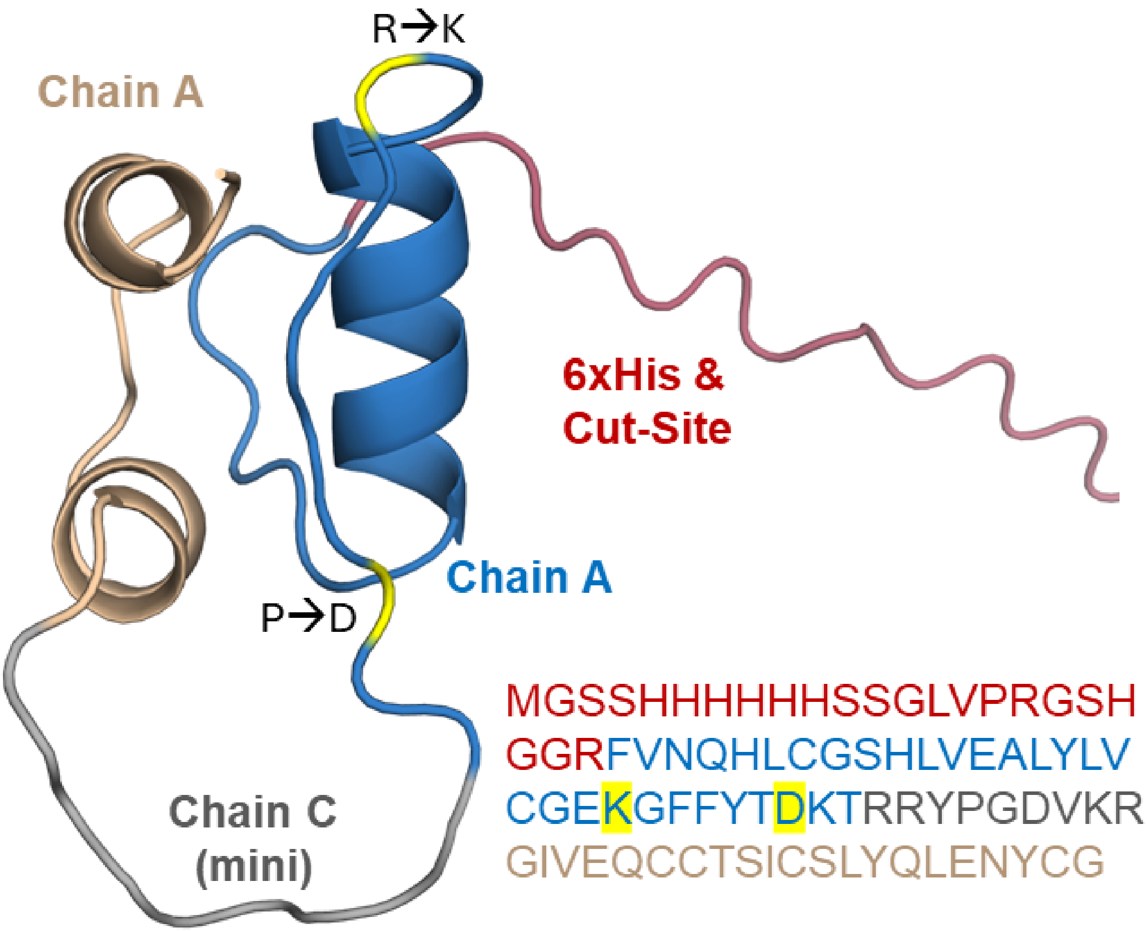

S1 Fig. Alpha-fold prediction of novel designer fast-acting mini-insulin and detailed sequence. Insulin has three domains: B-peptide (blue), C-peptide (gray), and A-peptide (orange). This design covers mini-modified C-peptide (YPGDV alone) as well as substitutions of RB22 to KB22 and PB28 to DB28 residues, which are highlighted in yellow. 6 x His tag and thrombin cut site are colored in red.
