# Supplementary material for: Optimizing recombinant mini proinsulin production via response surface method and microbioreactor screening
Source: PLoS One. 2025 Sep 8;20(9):e0329319. doi: 10.1371/journal.pone.0329319 (PMC12416663; doi:10.1371/journal.pone.0329319)
Supplement: S2 Fig — (PDF) [file pone.0329319.s002.pdf]

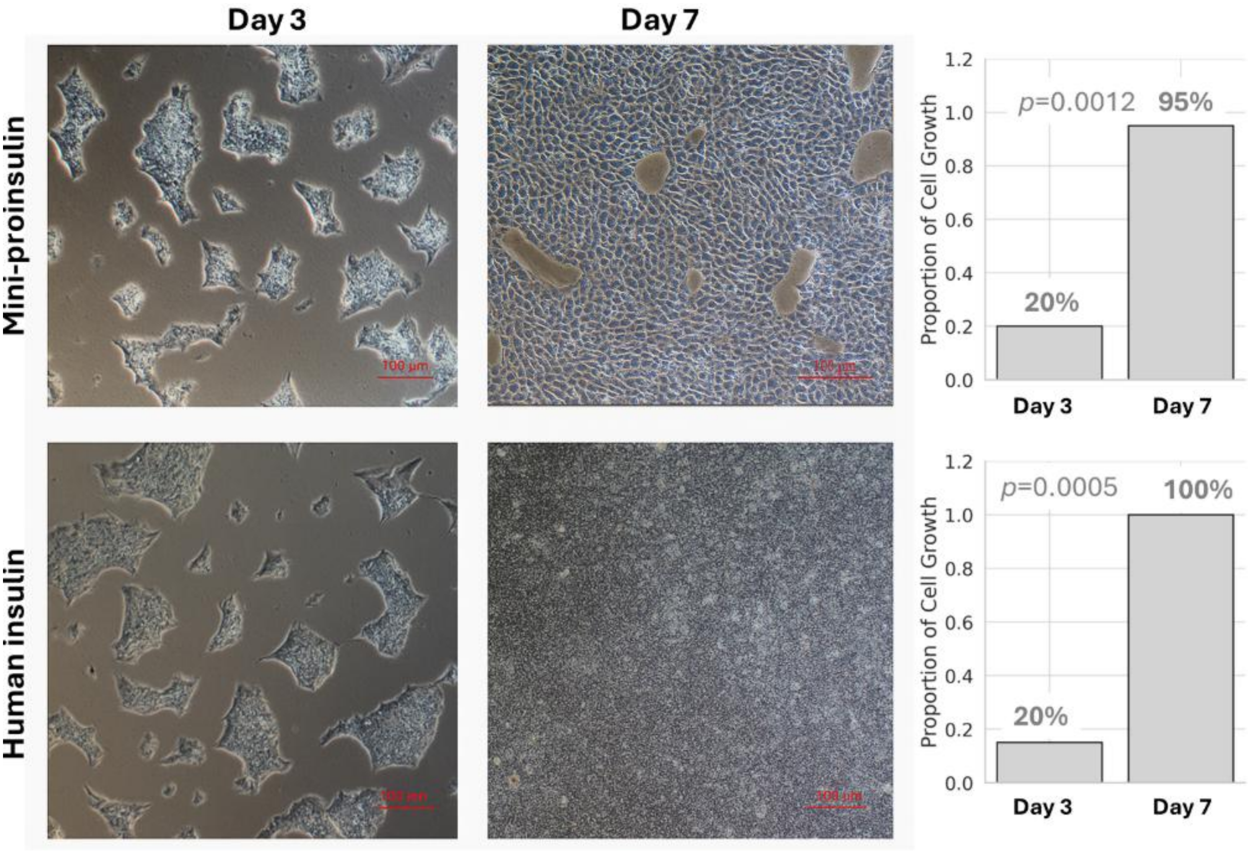

S2 Fig. Comparison of cell growth responses in hiPSCs treated with mini-proinsulin (nMPI) and human insulin over time. Phase-contrast microscopy images of human-induced pluripotent stem cells (hiPSCs) treated with nMPI (top row) or human insulin (bottom row) are shown at Day 3 and Day 7. Scale bars represent 100 µm. Cells treated with either insulin form exhibited increased confluency over time, with a marked difference between Day 3 and Day 7. Quantitative analysis of cell growth proportions is presented in the bar graphs (right panels), demonstrating a statistically significant increase for both nMPI ($p = 0.0012$) and human insulin ($p = 0.0005$) between Day 3 and Day 7. Human insulin induced complete confluency (100%) by Day 9, while nMPI also significantly promoted cell proliferation, reaching 95% confluency.
