# Supplementary material for: Optimizing recombinant mini proinsulin production via response surface method and microbioreactor screening
Source: PLoS One. 2025 Sep 8;20(9):e0329319. doi: 10.1371/journal.pone.0329319 (PMC12416663; doi:10.1371/journal.pone.0329319)
Supplement: S3 Fig — (PDF) [file pone.0329319.s003.pdf]

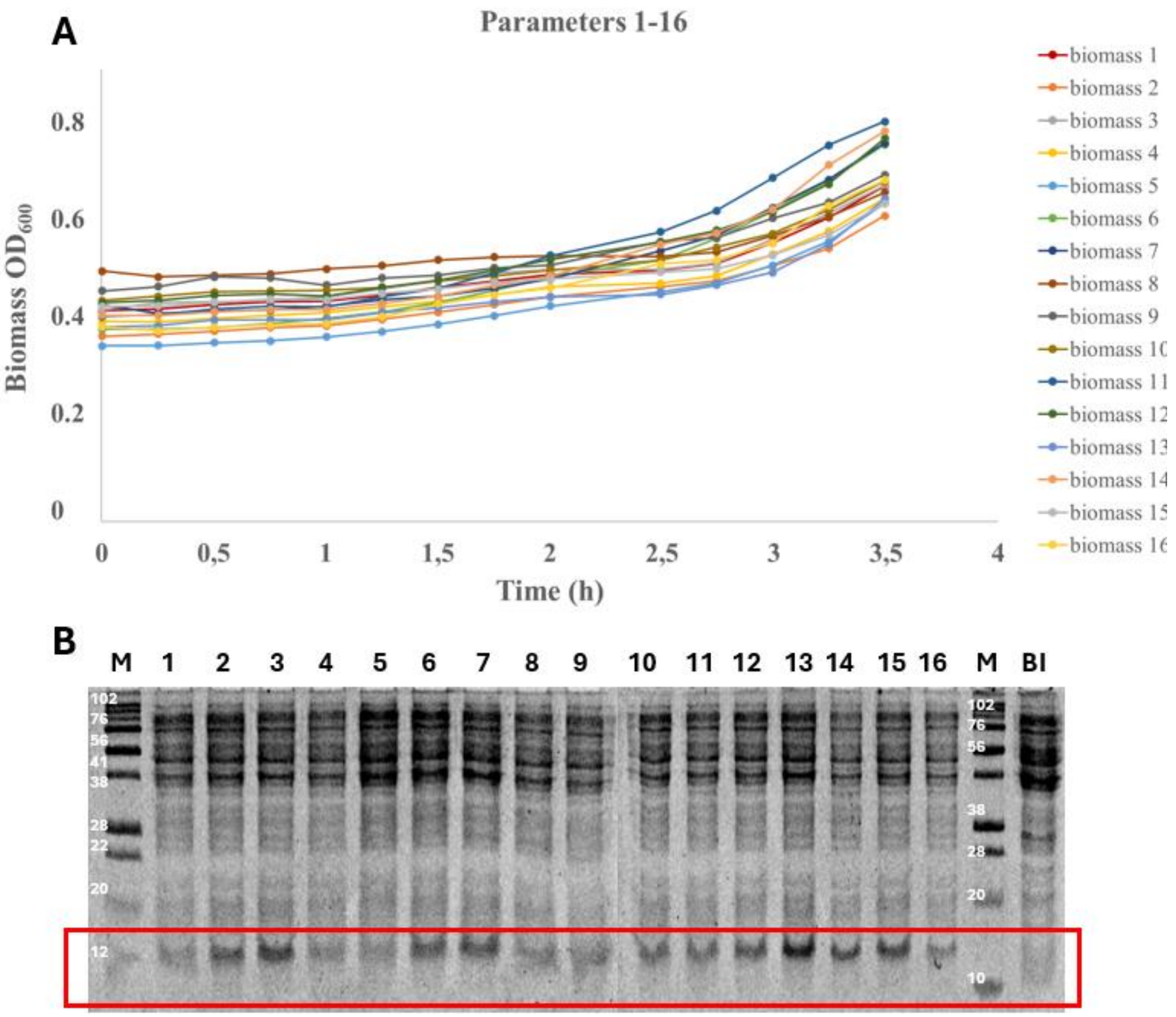

S3 Fig. Production efficacy of modified proinsulin and analytical characterization. **(A)** Growth kinetics of 16 parameters using the multi-well micro bioreactor. Each parameter was triplicated. **(B)** 20% SDS-PAGE analysis for determining insulin efficacy. Each band indicates each parameter's original protein yield, with no amount of equalization performed to assess direct protein efficacy. Produced insulin has been highlighted in red rectangle. M: marker, BI: before induction (notice that no insulin band before IPTG adding).
