# Supplementary material for: Optimizing recombinant mini proinsulin production via response surface method and microbioreactor screening
Source: PLoS One. 2025 Sep 8;20(9):e0329319. doi: 10.1371/journal.pone.0329319 (PMC12416663; doi:10.1371/journal.pone.0329319)
Supplement: S5 Fig — (PDF) [file pone.0329319.s005.pdf]

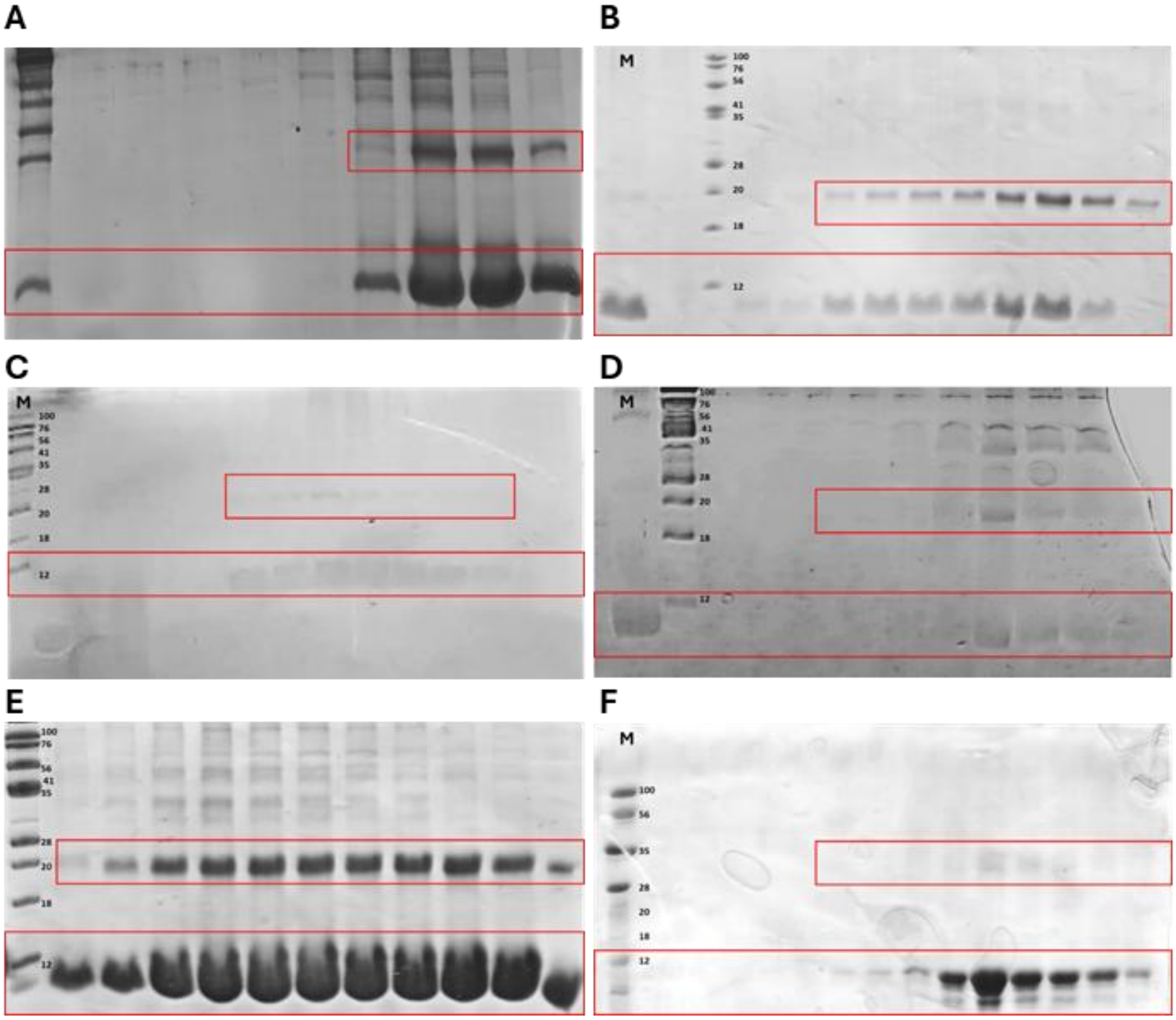

S5 Fig. Original 20% SDS-PAGE analysis of nMPI expression across microscale and large-scale cultivation conditions. (A) Expression profiles of nMPI under optimized Scenarios I–IV in the BioLector microbioreactor system. (B) Elution fractions (1–9) from nMPI production in 3-L bioreactor using LB Broth medium (30 g/L) as baseline control. (C–F) Elution fractions (1–9) from 3-L bioreactor cultivations under Scenario I (C), Scenario II (D), Scenario III (E), and Scenario IV (F), respectively. Red-highlighted bands in the 20–28 kDa range indicate potential nMPI oligomeric forms, while distinct bands near 12 kDa correspond to monomeric nMPI. The gel images reflect expression levels and purity across tested conditions, enabling direct comparison of scalability and media performance.
