# Supplementary material for: Optimizing recombinant mini proinsulin production via response surface method and microbioreactor screening
Source: PLoS One. 2025 Sep 8;20(9):e0329319. doi: 10.1371/journal.pone.0329319 (PMC12416663; doi:10.1371/journal.pone.0329319)
Supplement: S1 Table — (PDF) [file pone.0329319.s006.pdf]

S1 Table. The 16 parameters created by PBD analysis of eleven factors and insulin efficiencies were obtained due to experimental adaptation.

Std	Run	KCI (mM)	$MgCI_2$ (mM)	$MgSO_4$ (mM)	Glycerol (%)	Glucose (mM)	IPTG (mM)	pH	Yeast (g/L)	KH_2PO_4 (mM)	Na_2HPO_4 (mM)	Tiamine (mM)	Response : Insulin (g/L)
**11**	**1**	10	0	15	5	15	0,2	6,8	2,5	15	0	10	2,88
**14**	**2**	5	7,5	7,5	2,5	7,5	0,3	6,9	5	7,5	7,5	5	3,35
**15**	**3**	5	7,5	7,5	2,5	7,5	0,3	6,9	2,5	7,5	7,5	5	3,38
**13**	**4**	5	7,5	7,5	2,5	7,5	0,3	6,9	2,5	7,5	7,5	5	3,44
**5**	**5**	0	0	15	0	15	0,4	6,8	5	15	15	0	3,82
**2**	**6**	0	15	15	0	15	0,4	7	2,5	0	0	10	3,29
**9**	**7**	10	15	15	0	0	0,2	7	2,5	15	15	0	3,49
**1**	**8**	10	15	0	5	15	0,4	6,8	2,5	0	15	0	3,68
**4**	**9**	0	15	0	5	15	0,2	7	5	15	0	0	3,32
**8**	**10**	10	15	0	0	0	0,4	6,8	5	15	0	10	4,75
**12**	**11**	0	0	0	0	0	0,2	6,8	2,5	0	0	0	4,45
**7**	**12**	10	0	0	0	15	0,2	7	5	0	15	10	4,47
**10**	**13**	0	15	15	5	0	0,2	6,8	5	0	15	10	3,86
**3**	**14**	10	0	15	5	0	0,4	7	5	0	0	0	3,56
**6**	**15**	0	0	0	5	0	0,4	7	2,5	15	15	10	3,91
**16**	**16**	5	7,5	7,5	2,5	7,5	0,3	6,9	5	7,5	7,5	5	4,44
