# Supplementary material for: Optimizing recombinant mini proinsulin production via response surface method and microbioreactor screening
Source: PLoS One. 2025 Sep 8;20(9):e0329319. doi: 10.1371/journal.pone.0329319 (PMC12416663; doi:10.1371/journal.pone.0329319)
Supplement: S2 Table — (PDF) [file pone.0329319.s007.pdf]

S2 Table. Multi-well micro bioreactor parameters for achieving the highly-yielded biomass in a shorten time.

**Common Parameters**	
Microplate	MTP_48 FlowerPlate
Layout	48MTP
Lot	2102101
**Measurement Channels**	
Filter-1	Biomass
Filter-2	pH
Filter-3	Dissolved oxygen (DO)
**Measurement Parameters**	
Cycle time	15-min
Exp. time	Manual
**Environment**	
Enable Humidity Control	85 %
Temperature before induction	37 C
Temperature before induction	37 C
Shaking frequency	800 rpm
Enable O_2 Regulation	30 % O_2
