# Supplementary material for: Optimizing recombinant mini proinsulin production via response surface method and microbioreactor screening
Source: PLoS One. 2025 Sep 8;20(9):e0329319. doi: 10.1371/journal.pone.0329319 (PMC12416663; doi:10.1371/journal.pone.0329319)
Supplement: S3 Table — (PDF) [file pone.0329319.s008.pdf]

S3 Table. Coded and actual values of independent variables.

**Final Equation in Terms of Coded Factors**		**Final Equation in Terms of Actual Factors**	
**Insulin**	+379.00	**Insulin**	+439.16667
**MgSO4**	-30.67	**MgSO4**	-4.08889
**Glycerol**	-25.50	**Glycerol**	-10.20000
**Glucose**	-21.33	**Glucose**	-2.84444
**Yeast**	+17.33	**Yeast**	+34.66667
