# Supplementary material for: Optimizing recombinant mini proinsulin production via response surface method and microbioreactor screening
Source: PLoS One. 2025 Sep 8;20(9):e0329319. doi: 10.1371/journal.pone.0329319 (PMC12416663; doi:10.1371/journal.pone.0329319)
Supplement: S4 Table — (PDF) [file pone.0329319.s009.pdf]

S4 Table. The 30 parameters created by CCD analysis of statistically significant factors and insulin efficiencies were obtained due to experimental adaptation.

std	run	KCI (mM)	MgCI2 (mM)	KH2PO4 (mM)	Tiamine (mM)	IPTG (mM)	pH	Glucose (mM)	Glycerol (%)	Yeast (g/L)	MgSO4 (mM)	Insulin (g/L)
6	1	10	15	15	10	0,25	6,8	15	1	5	5	6,4
26	2	10	15	15	10	0,25	6,8	10	3	3,5	10	9,5
19	3	10	15	15	10	0,25	6,8	10	0,17	3,5	10	6,3
4	4	10	15	15	10	0,25	6,8	15	5	2,5	5	11
14	5	10	15	15	10	0,25	6,8	15	1	5	15	4,2
30	6	10	15	15	10	0,25	6,8	10	3	3,5	10	3,5
1	7	10	15	15	10	0,25	6,8	5	1	2,5	5	6
24	8	10	15	15	10	0,25	6,8	10	3	3,5	17,07	5,7
17	9	10	15	15	10	0,25	6,8	2,93	3	3,5	10	7,7
12	10	10	15	15	10	0,25	6,8	15	5	2,5	15	5
7	11	10	15	15	10	0,25	6,8	5	5	5	5	12,5
15	12	10	15	15	10	0,25	6,8	5	5	5	15	7,4
5	13	10	15	15	10	0,25	6,8	5	1	5	5	9,9
28	14	10	15	15	10	0,25	6,8	10	3	3,5	10	5,8
29	15	10	15	15	10	0,25	6,8	10	3	3,5	10	9
27	16	10	15	15	10	0,25	6,8	10	3	3,5	10	3,9
11	17	10	15	15	10	0,25	6,8	5	5	2,5	15	1,2
10	18	10	15	15	10	0,25	6,8	15	1	2,5	15	12,2
20	19	10	15	15	10	0,25	6,8	10	5,83	3,5	10	9,2
23	20	10	15	15	10	0,25	6,8	10	3	3,5	2,93	6,5
25	21	10	15	15	10	0,25	6,8	10	3	3,5	10	6,9
18	22	10	15	15	10	0,25	6,8	17,07	3	3,5	10	2,7
22	23	10	15	15	10	0,25	6,8	10	3	5,414	10	5
9	24	10	15	15	10	0,25	6,8	5	1	2,5	15	5,1
2	25	10	15	15	10	0,25	6,8	15	1	2,5	5	11,5
16	26	10	15	15	10	0,25	6,8	15	5	5	15	5,1
3	27	10	15	15	10	0,25	6,8	5	5	2,5	5	4,9
13	28	10	15	15	10	0,25	6,8	5	1	5	15	4
21	29	10	15	15	10	0,25	6,8	10	3	2,26	10	5,7
8	30	10	15	15	10	0,25	6,8	15	5	5	5	4,9
