# Supplementary material for: Optimizing recombinant mini proinsulin production via response surface method and microbioreactor screening
Source: PLoS One. 2025 Sep 8;20(9):e0329319. doi: 10.1371/journal.pone.0329319 (PMC12416663; doi:10.1371/journal.pone.0329319)
Supplement: S5 Table — (PDF) [file pone.0329319.s010.pdf]

S5 Table. Coded values of independent variables

Final Equation in Terms of Coded Factors	
**Insulin**	+ 14.59884
**Glucose**	+0.63756
**Glycerol**	-5.09749
**Yeast**	-0.034402
**$MgSO_4$**	+0.24010
**Glucose*Glycerol**	-0.067500
**Glucose*Yeast**	-8.00000E-003
**Glucose*$MgSO_4$**	-0.016500
**Glycerol*Yeast**	+0.025625
**Glycerol*$MgSO_4$**	-0.055000
**Glycerol*Glycerol**	+0.70312
