# Supplementary material for: Optimizing recombinant mini proinsulin production via response surface method and microbioreactor screening
Source: PLoS One. 2025 Sep 8;20(9):e0329319. doi: 10.1371/journal.pone.0329319 (PMC12416663; doi:10.1371/journal.pone.0329319)
Supplement: S1 Raw Images — (PDF) [file pone.0329319.s011.pdf]

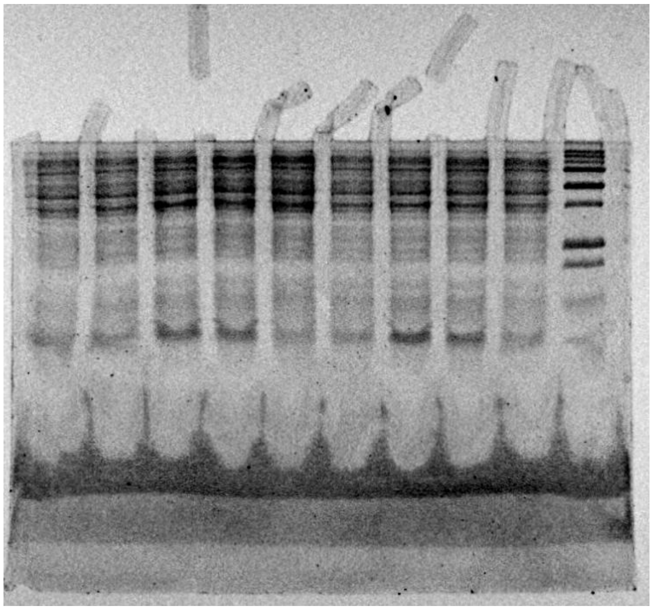

Raw image 1. Uncropped and unadjusted gel image of supplementary figure 3. Toward left: Marker and samples 1-9.

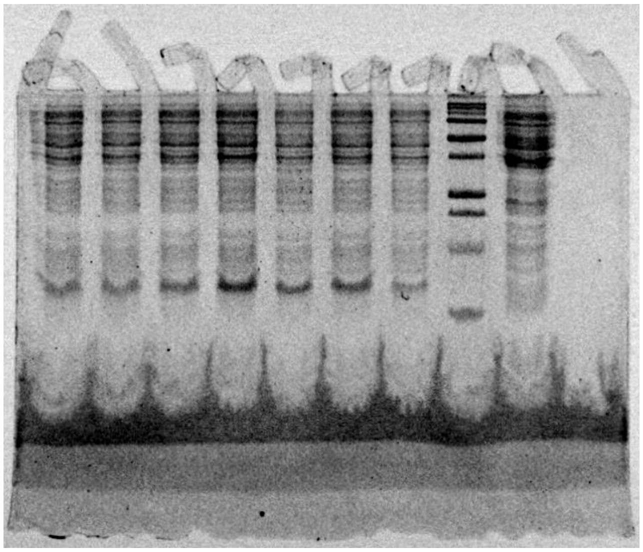

Raw image 2. Uncropped and unadjusted gel image of supplementary figure 3. Toward right: samples 10-16, marker, and before induction of insulin sample.

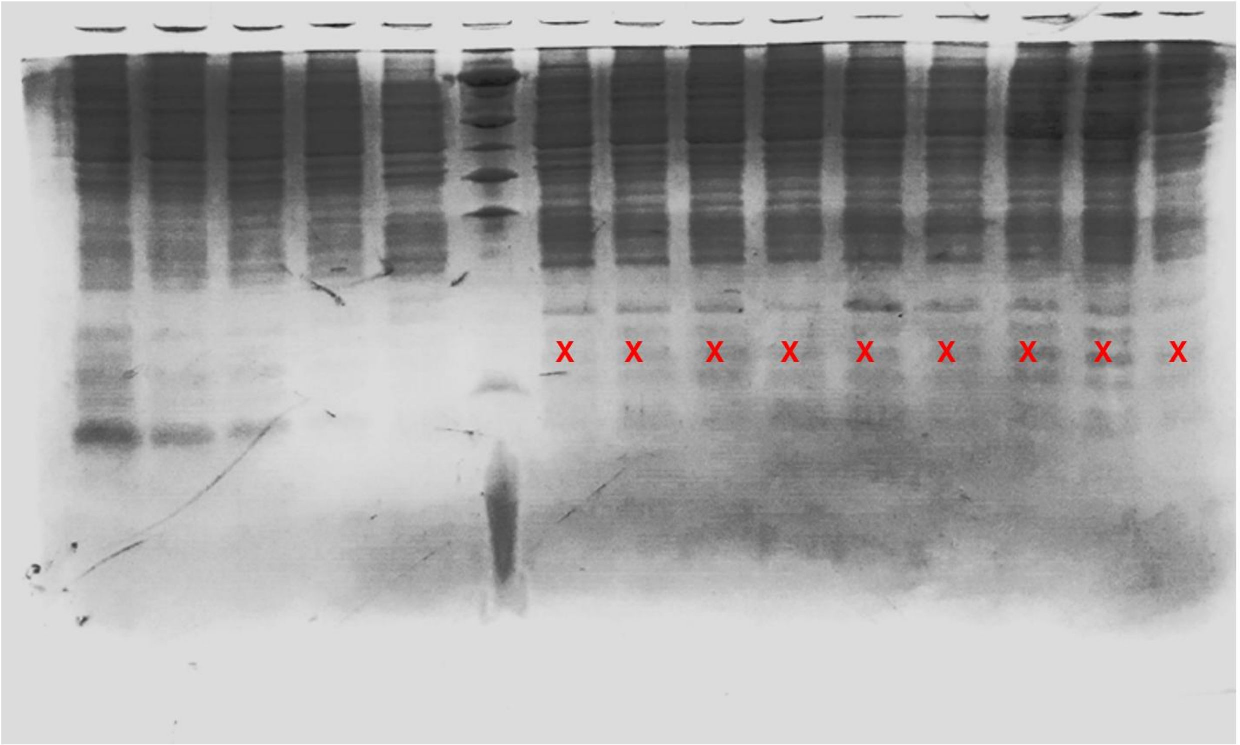

Raw image 3. Uncropped and unadjusted gel image of Box 1 in supplementary figure 4. Toward the right: samples 1-3, before induction of insulin in 2 wells respectively, marker. Wells that are not used in this study are shown with red X.

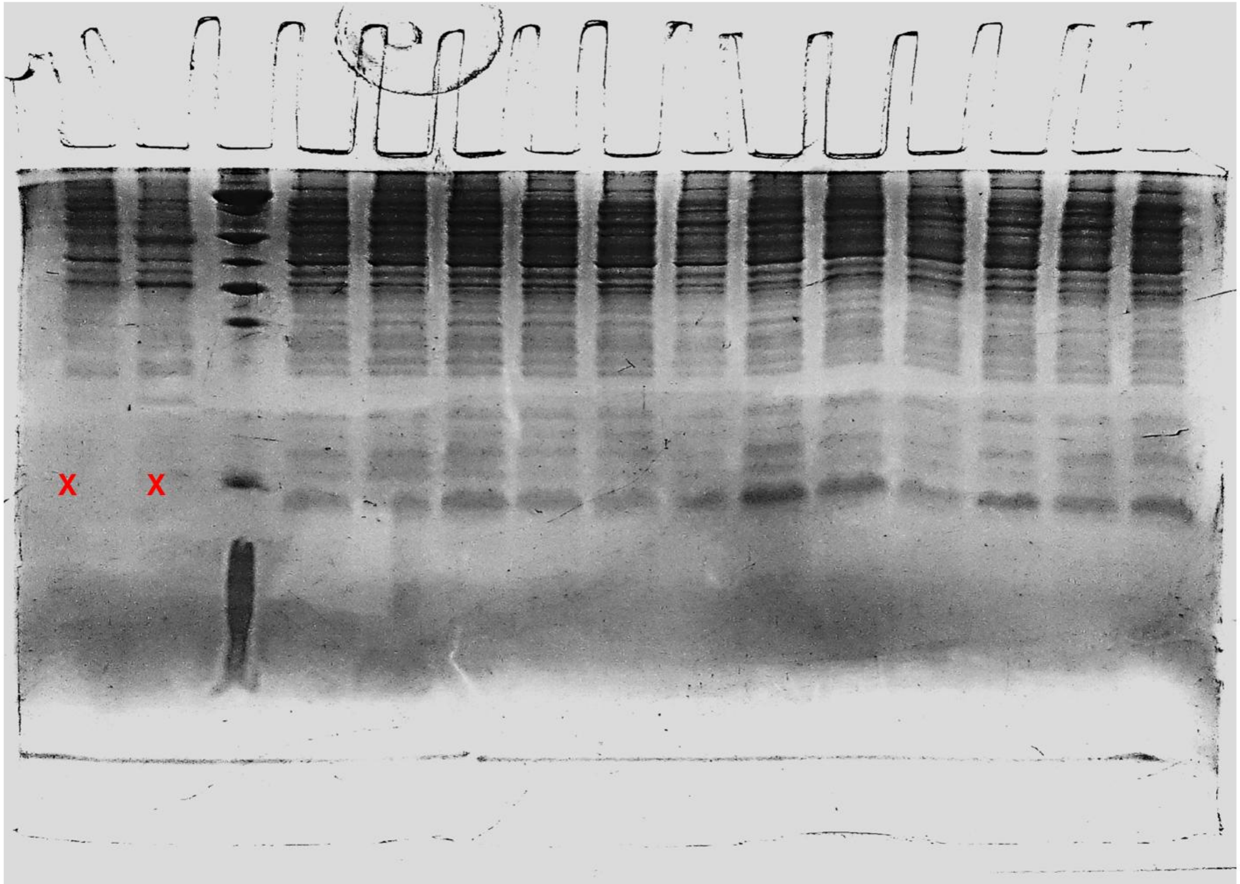

Raw image 4. Uncropped and unadjusted gel image of Box 2 in supplementary figure 4. Toward the right after useless wells: marker, samples 4-15. Wells that are not used in this study are shown with red X.

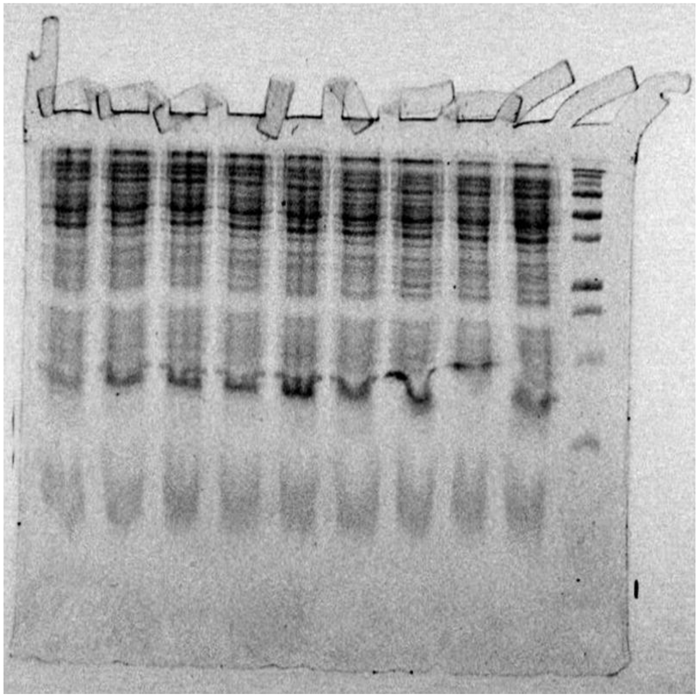

Raw image 5. Uncropped and unadjusted gel image of Box 3 in supplementary figure 4. Toward the right: samples 16-23 and marker, respectively.

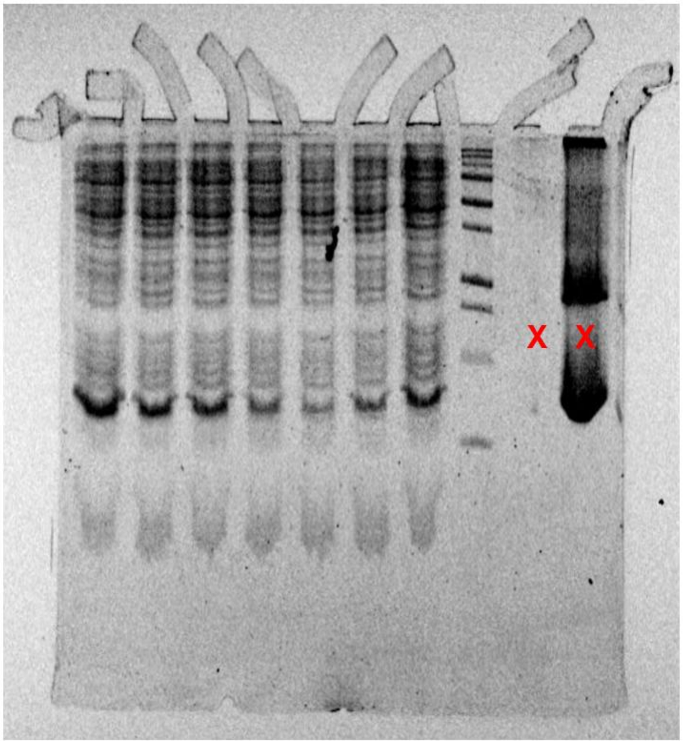

Raw image 6. Uncropped and unadjusted gel image of Box 4 in supplementary figure 4. Toward the right: samples 24-30 and marker, respectively. Wells that are not used in this study are shown with red X.

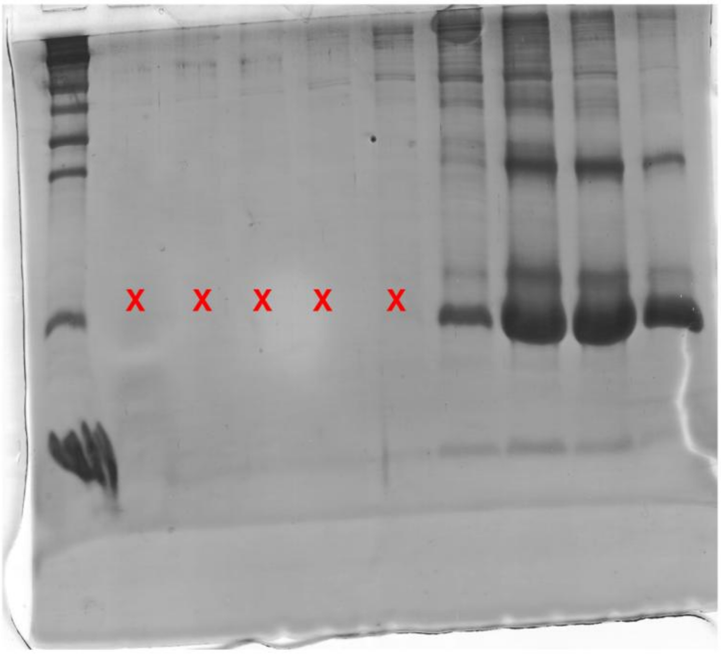

Raw image 7. Uncropped and unadjusted gel image of figure 6. Toward the right: marker, wells that are not used in this study, scenario 1, 3, 4, and 2., respectively. Wells that are not used in this study are shown with red X.

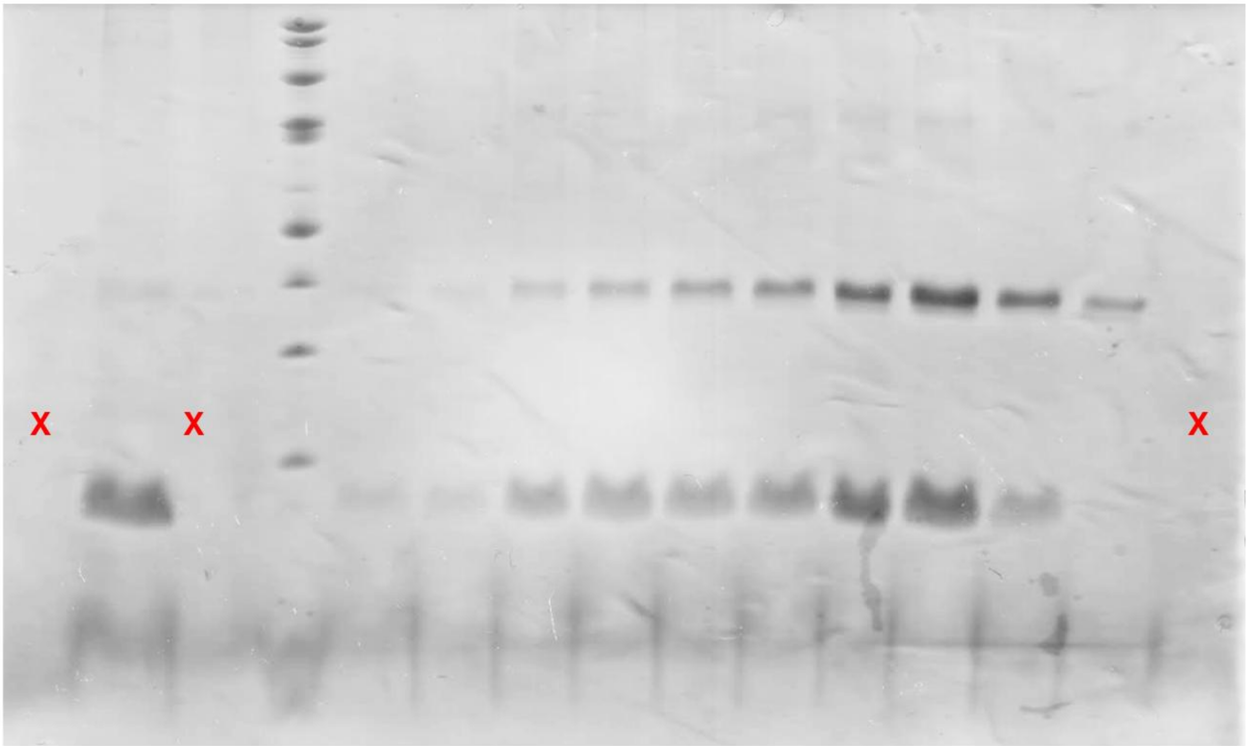

Raw image 8. Uncropped and unadjusted gel image of figure 7 and supplementary figure 5 (panel B). Toward the right: useless well, before SEC, useless well, marker, fractions 1-10, useless well, respectively. Wells that are not used in this study are shown with red X.

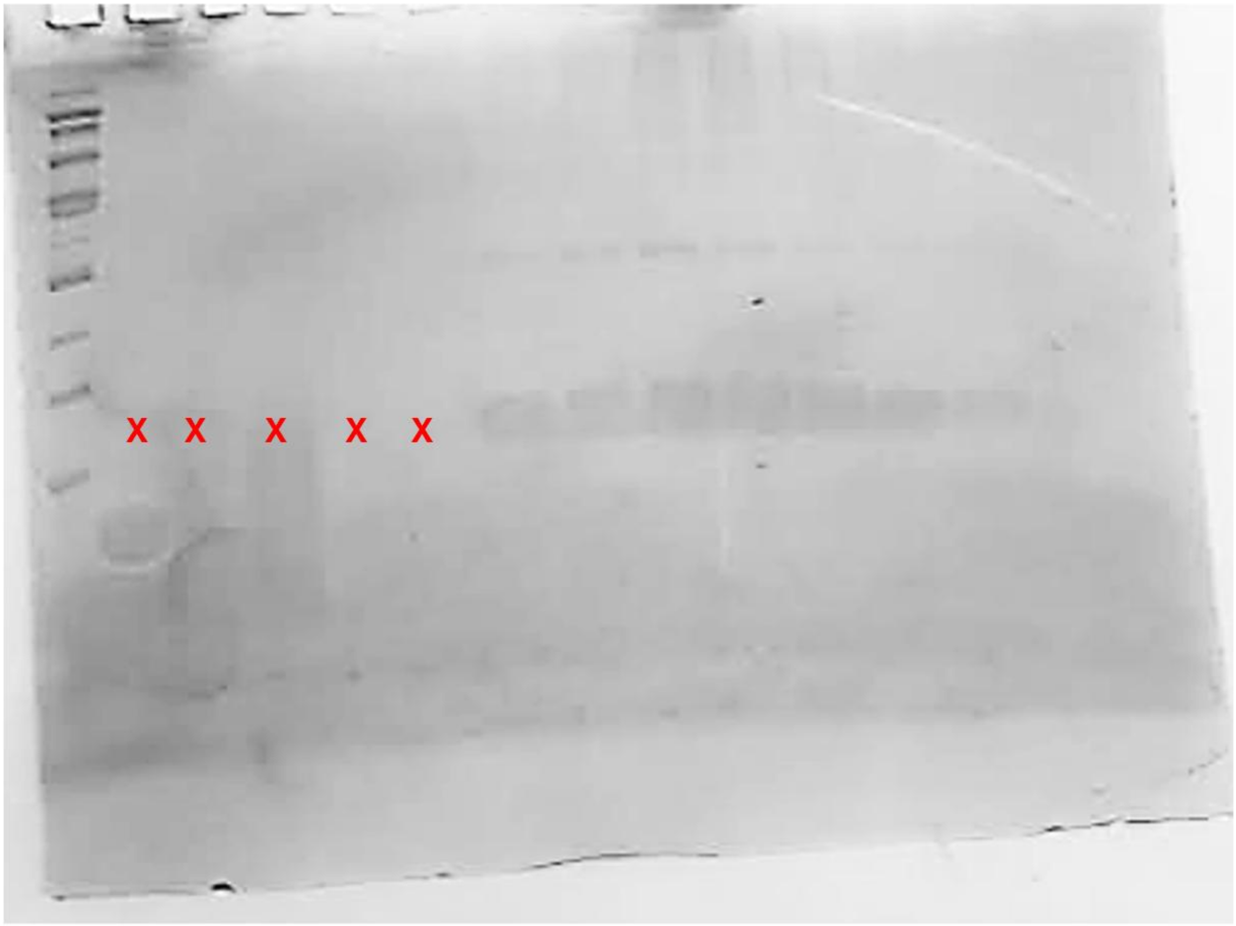

Raw image 9. Uncropped and unadjusted gel image of figure 7 (panel A) and supplementary figure 5 (panel C). Toward the right: marker, wells 2-5 that are not used in this study, fractions 1-8. Wells that are not used in this study are shown with red X.

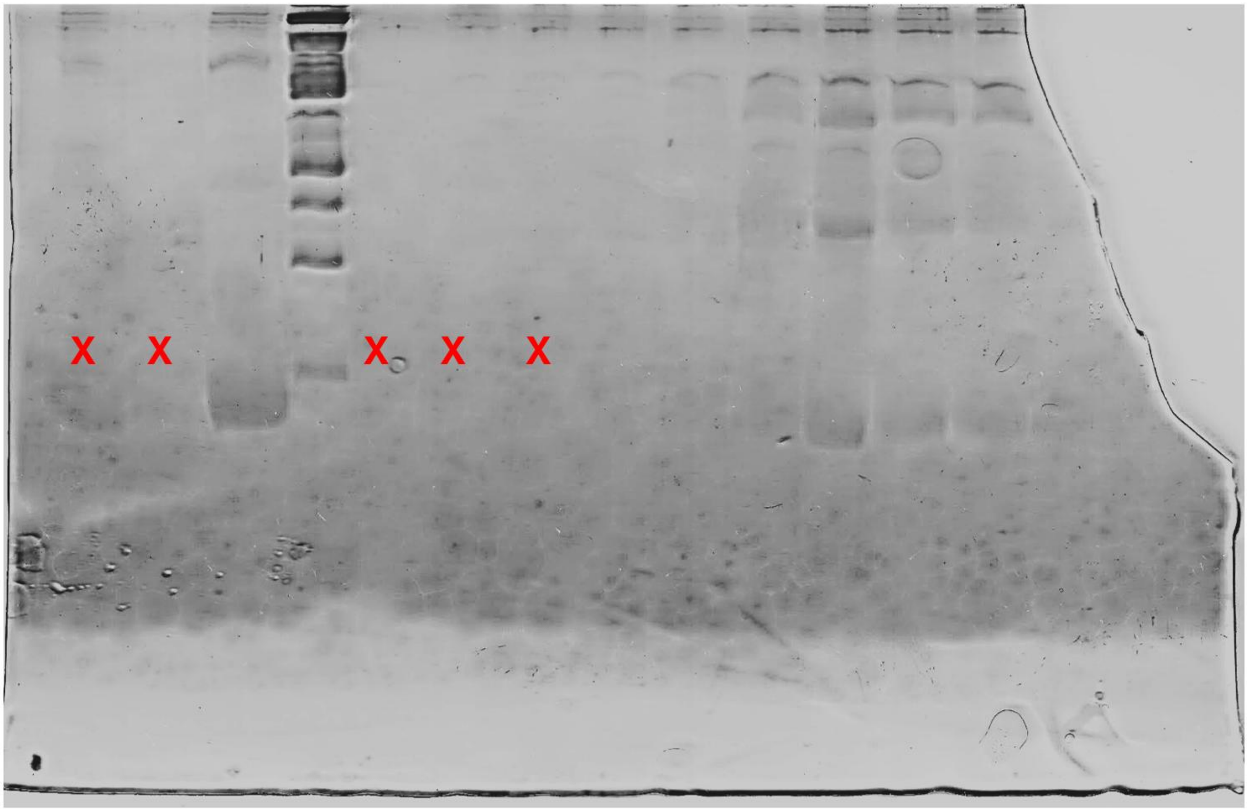

Raw image 10. Uncropped and unadjusted gel image of figure 7 (panel B) and supplementary figure 5 (panel D). Toward the right: wells 1-2 that are not used in this study, before SEC, marker, wells 5-7 that are not used in this study, fractions 1-9, respectively. Wells that are not used in this study are shown with red X.

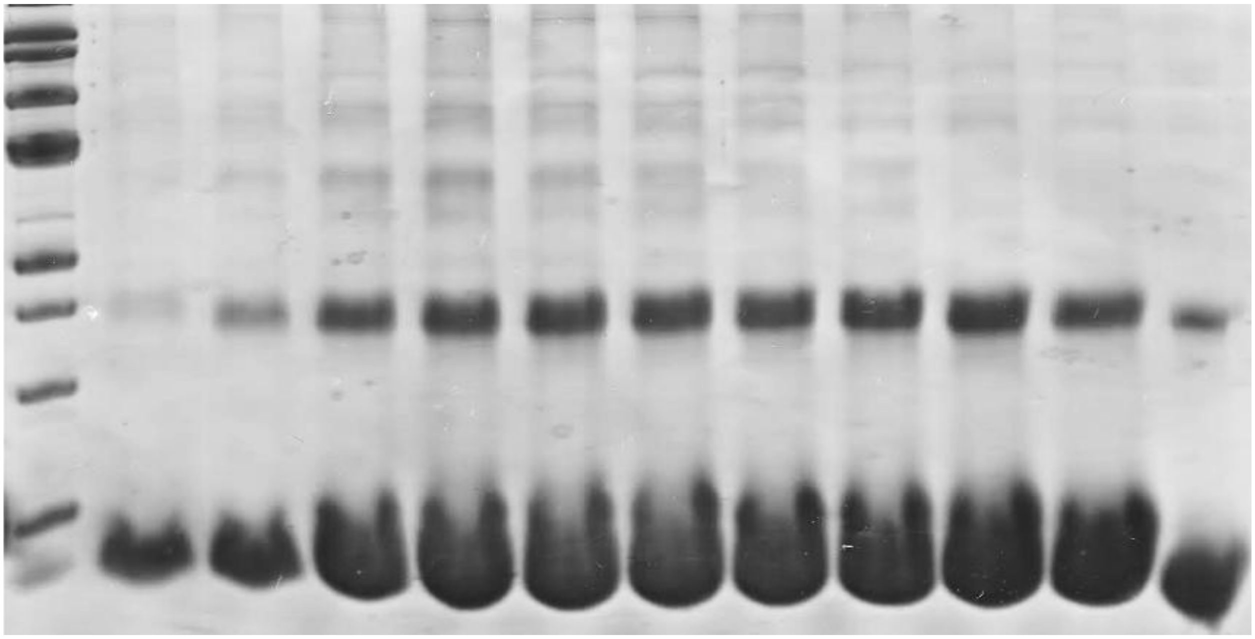

Raw image 11. Uncropped and unadjusted gel image of figure 7 (panel C) and supplementary figure 5 (panel E). Toward the right: marker and fractions 1-11.

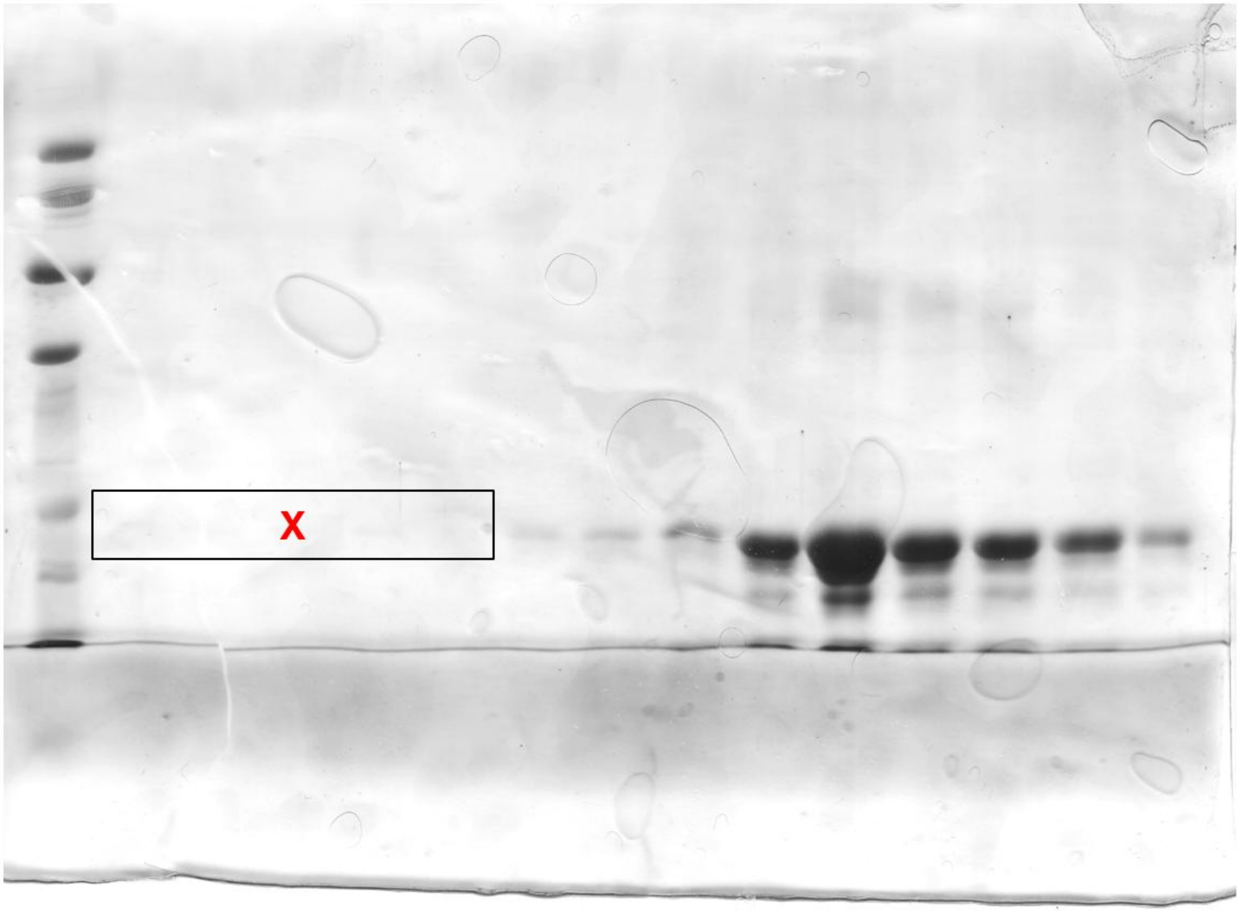

Raw image 12. Uncropped and unadjusted gel image of figure 7 (panel D) and supplementary figure 5 (panel F). Toward the right: marker, wells 2-6 that are not used in this study, fractions 7-15. Wells that are not used in this study are shown with red X.
